# A cellular hierarchy of Notch and Kras signaling controls cell fate specification in the developing mouse salivary gland

**DOI:** 10.1016/j.devcel.2022.12.009

**Published:** 2023-01-23

**Authors:** Lemonia Chatzeli, Ignacio Bordeu, Seungmin Han, Sara Bisetto, Zahra Waheed, Maria P. Alcolea, Benjamin D. Simons

**Affiliations:** 1 Wellcome Trust/Cancer Research UK Gurdon Institute, University of Cambridge, Cambridge, CB2 1QN, UK; 2 Wellcome Trust-Medical Research Council Cambridge Stem Cell Institute, Jeffrey Cheah Biomedical Centre, University of Cambridge, Cambridge, CB2 0AW, UK; 3 Department of Applied Mathematics and Theoretical Physics, Centre for Mathematical Sciences, University of Cambridge, Cambridge, CB3 0WA, UK; 4 Departamento de Física, Facultad de Ciencias Físicas y Matemáticas, Universidad de Chile, Santiago, 837.0415, Chile

**Keywords:** salivary gland, branching, differentiation, potency, Kras, Notch, development, duct, acini, Krt14

## Abstract

The development of the mouse salivary gland involves a tip-driven process of branching morphogenesis that takes place in concert with differentiation into acinar, myoepithelial and ductal (basal and luminal) sub-lineages. By combining clonal lineage tracing with 3D reconstruction of the branched epithelial network and single-cell RNA-seq analysis, we show that in tips a heterogeneous population of renewing progenitors transition from a Krt14+ multipotent state to unipotent states via two transcriptionally distinct bipotent states, one restricted to the Krt14+ basal and myoepithelial lineage, and the other to the Krt8+ acinar and luminal lineage. Using genetic perturbations, we show how differential expression of Notch signalling correlates with spatial segregation, exit from multipotency and promotion of the Krt8+ lineage, while Kras activation promotes proacinar fate. These findings provide a mechanistic basis for how positional cues within growing tips regulate the process of lineage segregation and ductal patterning.

## Introduction

Head and neck cancer accounts for up to 5% of cancers worldwide. ^1^ Most of these patients undergo radiation treatment, which has a deleterious effect on salivary gland (SG) anatomy and function, leading to xerostomia. ^2^ To relieve such side effects, there is increasing interest in strategies to regenerate SGs. ^3^ This requires the identification of the progenitors, signaling pathways and programs involved in lineage specification and patterning. ^4,5^ In mammals, the major SGs comprise 3 distinct gland pairs: the parotid, sublingual, and submandibular gland (SMG) (Figure S1A) (Miletich, 2010). Among them the SMG is the most heavily studied and is the focus here.

In mouse, the SMG initiates at embryonic day (E)11.5 when the epithelium invaginates into the mesenchyme creating a placode. At E12.5, the epithelium forms a stalk that terminates in an endbud (initial bud stage). The endbud then undergoes cleft formation and elongation to generate secondary ducts (E13.5). Subsequently, serial rounds of cleft formation, endbud branching and ductal elongation lead to a complex ductal network. During this process, the multilayered structure of endbuds transforms progressively into a secretory unit (terminal bud stage at E18.5) by which time the 3 main SG cell types are established: ductal cells (basal and luminal), saliva-producing acinar cells, and myoepithelial cells that surround the acini and aid secretion (Figure S1B). Terminal maturation of acini and ducts proceeds postnatally. ^6–8^

To understand how progenitors restrict their fate and transition from a primitive multilayer placode to a branched organ with acinar, ductal and myoepithelial identity, studies have focused on population-based lineage tracing strategies (reviewed in Aure et al. ^9^). Conditional lineage tracing with promoters expressed initially at the placode and later at the endbud of the initial bud stage (E12.5), using *Sox9creERT*, ^10^ *Trp63creERT*, ^11^ *Krt14creERT*, ^12^ and *Sox10creERT* ^13^ mouse lines, leads to labelling across the entire branching epithelium, suggesting that the whole gland originates from these endbud progenitors. By contrast, conditional lineage tracing at E12-E13 based on Sox2, a promoter expressed in the stalk region of the initial bud in SMGs, generates exclusively cells of the main duct at E18.5, suggesting little or no contribution of early ductal cells to the formation of subsequent branches. ^13^ Further, tracing studies have shown that, as branching proceeds, endbud progenitors gradually restrict their fate. Krt14+ progenitors remain multipotent until E15.5, capable of generating all 4 cell types while, from E16.5, fate is restricted to ductal and myoepithelial lineages. ^12^

While population-based studies provide valuable insights into how changes in fate restriction correlate with molecular identity, they cannot resolve the lineage potential (potency), proliferative capacity, and multiplicity of individual progenitors, nor the timings of restriction. Based on existing studies, it remains unclear whether multipotency is a property of individual *Krt14+* cells or only the *Krt14*-expressing population as a whole. Does lineage restriction occur directly, through transition into unipotent states fated for one of the 4 lineages, or are progenitors organized hierarchically, with restriction occurring sequentially through distinct transitional states? Is lineage restriction coordinated uniformly across the gland in response to systemic cues, or does it occur progressively in response to local cues and at different stages between and/or within individual endbuds, as observed in the mouse pancreas and mammary gland? ^14,15^ Finally, which signaling factors drive lineage restriction, and how do they correlate with changes in spatial organization of progenitors?

To address factors governing the large-scale organization of the SG epithelium, we recently mapped quantitatively in 3D the developing organ (Bordeu et al., unpublished). Using a statistical modelling-based approach, these studies showed that, after an initial phase of endbud diversification, the network topology develops as a stochastic branching process in which progenitors positioned at endbuds renew, driving rounds of tip bifurcation and ductal elongation, similar to that observed in the development of the mouse mammary gland epithelium. ^16–18^ However, in contrast to mammary ductal morphogenesis, where tip growth is terminated by signals from proximate ducts, endbuds in the SG are only delayed by steric or biochemical influences, with the branching process continuing as constraints become alleviated through the expansion of the mesenchyme (Figure S1C). These findings suggest a morphogenic program in which the development of the large-scale branching organization may be decoupled from the potency of progenitors. Here, by tracing individual cell lineages, we define the identity, multiplicity, and potency of the progenitor populations during embryonic development. Further, by combining these findings with single-cell profiling, we use functional assays to explore the role of specific signaling pathways in enabling tip cells to control the balance between lineage selection.

## Results

### Endbud progenitors undergo early and progressive lineage restriction

**Figure 1:**
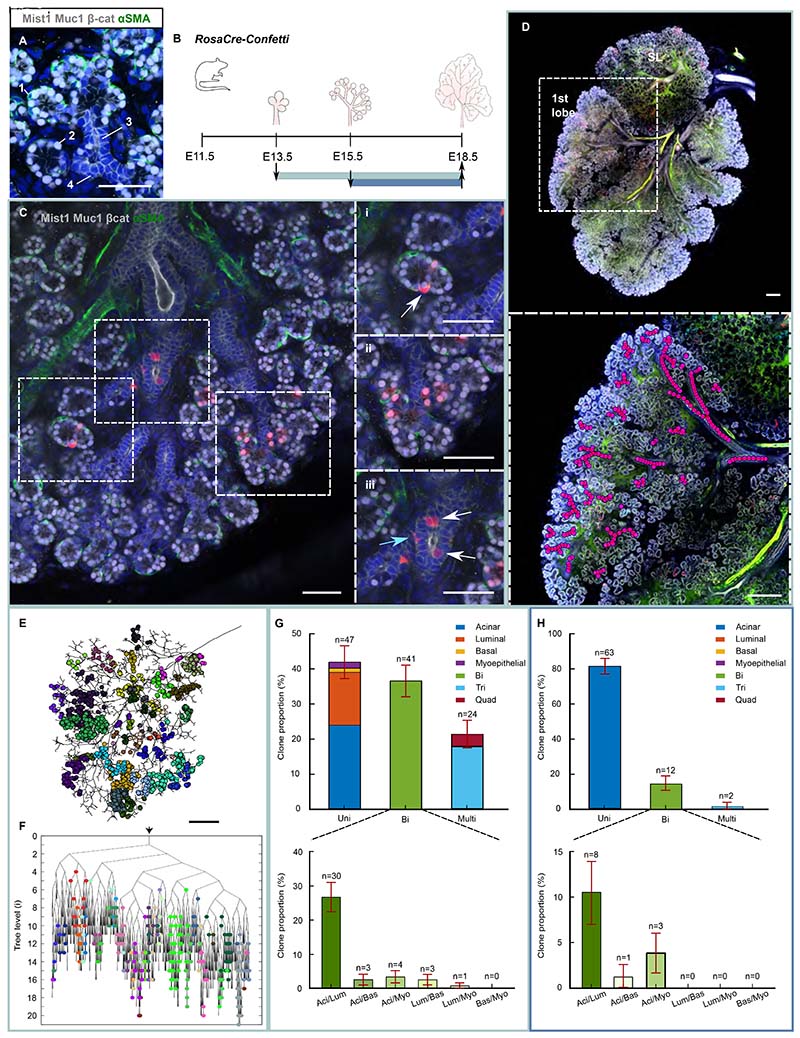
Endbud progenitors undergo early and progressive lineage restriction. (A) Identification of myoepithelial (1) (cytoplasmic αSMA: green), acinar (2) (nuclear Mist1 and cytoplasmic β-cat: grey), luminal (3) (cytoplasmic β-cat and apical Mucin 1: grey and negative nuclear Mist1) and basal (4) (cytoplasmic β-cat: grey and negative nuclear Mist1) cells in E18.5 SMG. DAPI: blue. Scale bar: 50μm. (B) *RosaCre-Confetti* mice were clonally induced at E13.5 (green line) or E15.5 (blue) and SMGs collected at E18.5. (C) Multipotent RFP clone from E13.5-E18.5 tracing (green bounding box). Magnified image of C with arrow pointing at (i) RFP myoepithelial cell, (ii) acinar cells, and (iii) a basal ductal (blue arrow) and luminal ductal cell (white arrow). Scale bars: 50μm. See Movie S2. (D) Whole view image of E18.5 *RosaCre-Confetti* submandibular-sublingual gland induced at E13.5 (green bounding box) and stained as in A. Red: RFP, yellow: EYFP, cyan: mCFP, green: nGFP. SL: Sublingual. Box: Higher magnification image of first lobe in D used for clonal analysis and ductal reconstruction. Scale bars: 200μm. See Figure S1. (E) Skeletonized image of E18.5 ductal network with segmented clones induced at E13.5 (green bounding box). Clones indicated as circular markers. Scale bar: 200μm. See Movie S1. (F) Branching tree from E. See Figure S2A,B. Arrow at the top indicates the starting point. (G) Top: Proportion of uni-, bi- and multipotent clones (tri- and quadripotent) from *RosaCre-Confetti* mice traced from E13.5-E18.5 (green bounding box) ± standard error of proportion (SEP). Bottom: Proportion of bipotent clones ±SEP with combinations as listed. n=112 clones from 3 mice. See Table S1 and Methods. (H) Top: Proportion of uni-, bi- and multipotent clones (tri- and quadripotent) from *RosaCre-Confetti* mice traced from E15.5-E18.5 (blue bounding box) ±SEP. Bottom: Proportion of bipotent clones. n=77 clones from 3 mice. See Table S1 and Methods.

To study the potency of mouse SG progenitors, we first performed fate mapping and ductal reconstruction of embryonic SMGs at E18.5, when the main cell types are specified and the gross organization of the network is complete ^6^ (Figure 1 and Figure S1D-M, Figure S2). The SMG comprises multiple lobes, each of which has the capacity to develop independently into a complex ductal structure. ^19^ Therefore, to manually reconstruct the network, we analyzed a single lobe in each gland, choosing the lobe more proximal to the sublingual gland, hereafter referred to as lobe 1 (Figure 1A-D).

To perform an unbiased analysis, we first traced cells at clonal induction using the ubiquitously expressed *Rosa26-CreERT2/Rosa26-Confetti* mouse line (*RosaCreERT;Confetti*) (Figure 1B). Upon tamoxifen (TAM) administration, this system results in the random labelling of cells in one of 4 confetti colors (CFP, RFP, YFP and GFP) ^20^ (Figure S1D-M). TAM was delivered at E13.5 with cells becoming induced at ˜E14.5 (Figure S1D-M, Figure S3). At E14.5, a rudimentary tree is already formed in lobe 1 (comprising 4-15 generations of branching) (Figure S3A,B). To map individual clones onto the network at E18.5, we first generated 3D maps of lobe 1 and marked the coordinates of each labelled cell (Figure 1D-F, Movie S1, Movie S2 and Figure S2) (see Methods and discussion below). Two clone types could be identified: a minority of clones (12%; 15 out of 127) were restricted entirely within a single duct or acinus, while the majority (88%) spanned consecutive branch points and/or acini (Figure S2G). Since the latter must have derived from progenitors capable of multiplying during endbud duplication, we refer to these as “renewing”, i.e., clones rooted in progenitors with renewal or stem cell-like potential. In rare cases (Methods and Figure S3C-D), we identified individual acinar cells located within the luminal compartment of ducts. However, they were not present at the adult stage.

In line with the tissue composition, most constituent cells in clones at E18.5 were of acinar and luminal ductal type (Figure S3E). However, labelled cells of myoepithelial or basal identity were found to be underrepresented compared to tissue, suggesting that induction under the *RosaCreERT* promoter was not completely unbiased (Figure S3E). Notably, images acquired soon after induction at E14.5 showed that most labelled cells were located at the inner layers of the endbud, where proliferation is greater than the outer Krt14+ cell layer (Figure S3A,F,G). ^21^ As seen later, this positional bias in induction likely explains the small bias in composition. However, since our focus is on uncovering the repertoire of progenitor states, as well as the potency and proliferative potential of individual cells, we will see that this bias does not impact the analysis.

Focusing on renewing clones, we found that they could be comprised of cells from a single lineage (termed unipotent) or multiple lineages (termed bi- or multipotent depending on composition) (Figure 1G). Strikingly, from clonal reconstructions, we found that 42±5% (±SEP) of progenitors gave rise to cells belonging to either the acinar or luminal ductal compartment but not both, suggesting that lineage restriction may constitute an early event in SG development (Figure 1G). Such behavior stood in contrast with the timing of protein expression of the proacinar-specific differentiation markers Nkcc1 and Aqp5, which occur at E15.5. ^11,22–24^ Alongside unipotent clones, 37±5% (±SEP) of clones were bipotent, and 21±4% (±SEP) multipotent (Figure 1G).

To investigate whether potency changes during development, we traced endbud progenitors from E15.5-E18.5 using the *RosaCreERT;Confetti* model (Figure 1B,H). In this case, 82±4% (±SEP) of renewing clones were unipotent while only 2.6±1.8% (±SEP) were tripotent. Since, at this time point, 1-3% of clones were estimated to arise from chance merger (Methods), we could not rule out that some or all clones identified as tripotent could instead be associated with the merger of uni- and/or bipotent clones. Consistent with the sharp reduction in the generation of new ductal branches from E16.5-E18.5, combined with the massive expansion of the acinar compartment during this period, all unipotent clones were of acinar type (Figure 1H). Together, these observations are consistent with progressive lineage restriction of renewing tip progenitors. Moreover, since the range of potencies was found to be diverse at both induction times, we reasoned that changes in fate restriction are not synchronized across the gland, but likely linked to local factors, allowing progenitors to become fate restricted at different developmental times and stages.

### Renewing endbud progenitors are abundant, well mixed and segregate randomly during endbud bifurcation

**Figure 2:**
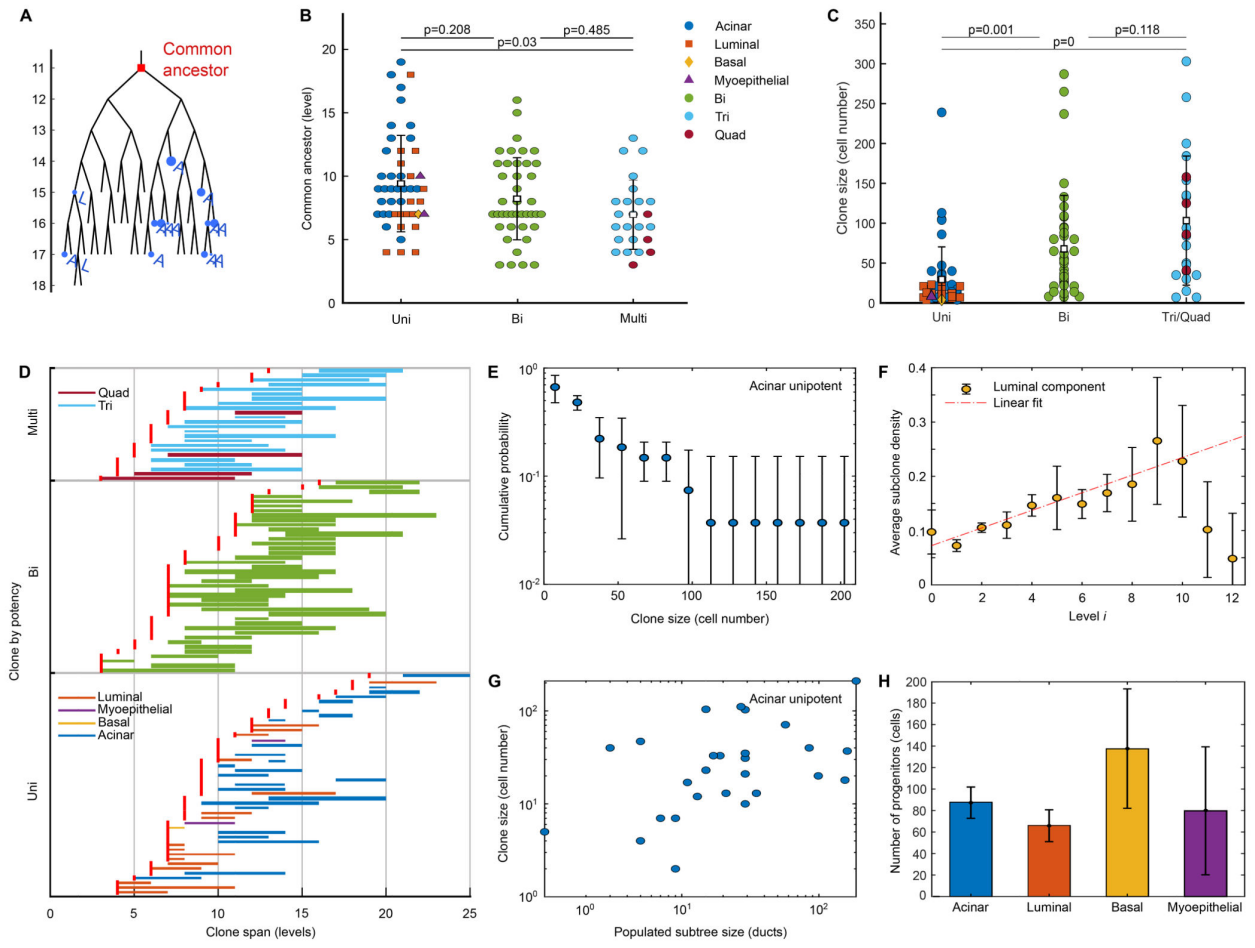
Renewing endbud progenitors are well mixed and disperse equally within sister branches. (A) Example of a clone mapped onto a subtree, showing position of common ancestor (red) and subclones (blue), together with corresponding cell type of each subclone: acinar (A) or luminal (L). (B) Level of common ancestor for clones containing a single (uni), two (bi) or >2 (multi) cell types (mean ±SD). Markers indicate identity. (C) Number of cells per clones containing a single (uni), two (bi) or >2 (multi) cell types (mean ±SD). Markers as B. See Methods. n=112 clones from 3 mice. (D) Clone span for uni, bi and multi cell type clones. Each horizontal line corresponds to a clone, beginning and ending at the levels of the first and last labelled cell in the tree, with the line width scaling with the number of cells in clone. For each clone, the red square indicates level of common ancestor. n=112 clones from 3 mice. See Table S2. (E) Cumulative distribution of acinar unipotent clones, where lines show fit to exponential decay ±SD. n=27 clones from 3 mice. (F) Average density ±SD of luminal subclones per duct as a function of level. Black line corresponds to a linear fit of first 18 generations. n = 3 mice. (G) Correlation between acinar unipotent clone size and subtree size. n=27 clones from 3 mice. (H) Estimated number of acinar, luminal, basal and myoepithelial progenitors based on average fractional contribution of progenitor types to subtrees (mean ±SD). See Table S3 and Figure S4.

To gain further insight into the pattern of lineage restriction, we took advantage of the nature of the branching process in which the identity of cells in trailing ducts provides a record of fate decisions at tips. ^14,16^ As a starting point, we used the positions of labelled cells within ductal trees to infer the likely position of the “common ancestor (CA)” at the time of induction. The CA was defined as the highest node in the tree from which stems the minimal subtree that contains the entirety of the clone (Figure 2A and Methods). We reasoned that a clone with a CA towards the apex of the tree (lower branch levels) was likely to be generated by a more primitive progenitor than a clone where the CA resides at higher levels. For each unipotent and multipotent clone we plotted the CA level and the maximal span across the tree (Figure 2B). Consistent with a gradual progression of lineage restriction, CAs of multipotent clones were found at lower levels (level 7 on average) than those of unipotent clones (level 9-10 on average) (Figure 2B and Figure S4A).

Clones displayed a broad distribution of sizes both between different lineages and within the same lineage (Figure 2C-E) that could populate a small or large fraction of the network. Clones with a lower CA were also more likely to populate larger fractions of the gland and thus expand to larger sizes (Figure 2D, Figure S4B and Methods).

To investigate how clones disperse across the network during rounds of branching, we considered how the average size of subclones, defined as the subset of ductal cells in a clone that occupy a single duct, change with distance (levels) from their CA. If renewing cells maintain their position across rounds of bifurcation, the average subclone size is predicted to expand exponentially with level number. ^16^ If, on the other hand, progenitors are “well-mixed” and randomly segregated during endbud bifurcation, the average subclone size expands only linearly with branch level. Applied to SG, as ducts in the tree decrease in average length with increasing level index (with the width remaining roughly constant) (Figure S4C), we first normalized subclone sizes by the duct length, providing a measure of the labelled cell density in subclones (Figure 2F). The results revealed a linear-like increase, mirroring the behavior found in mammary gland. This suggests a random partitioning of renewing progenitors between daughter endbuds, a result confirmed through simulation of the branching program (Figure S4D), and consistent with live-imaging studies demonstrating rapid movement of cells at endbuds. ^21,25,26^ Such dispersive behavior during tip bifurcation both validates *a posteriori* our association of the last CA as the likely origin of the clone and explains the statistical correlation between clone size and the size of the subtree they occupy (Figure 2G).

Previously, we found that multi-, bi- and unipotent clones coexist in the same gland. However, this does not determine whether progenitors of different potencies can coexist within the same endbud. From among the ensemble of clones, we found events where clones of different colors share the same CA and were therefore likely to coexist in the same endbud at induction. From n=3 animals, we found that 10 out of 112 CAs were associated with clones of more than one color. Among these occurrences, we found evidence for the coexistence of both multi- and bipotent clones with unipotent clones (Table S2), suggesting that lineage restriction is not coordinated synchronously across individual endbuds. Later, we consider how signaling factors and positional cues can instruct differential changes in the lineage potential of progenitors within the same endbud.

Based on this observation, we sought to estimate the abundance of renewing progenitors in individual endbuds. Do only a handful of cells maintain renewal potential, as found in mouse pancreas, or is it the majority, as observed in mammary gland? Using the average fractional contribution of progenitor types to subtrees to estimate the multiplicity of renewing cells (Figure 2H, Figure S4E, Table S3 and Methods), we found that most endbud progenitors harbor renewal potential.

Overall, these findings support a model in which renewing progenitors undergo progressive lineage restriction in a manner that is temporally overlapping both across and within individual endbuds. During bifurcation, progenitors amplify in number and their progenies are randomly segregated between daughter tips. ^21,25,27,28^ The clonal data suggested that the transition from a multi- to a unipotent acinar and luminal progenitor may proceed via an intermediate bipotent progenitor state. However, since the number of labelled myoepithelial and basal cells is low, it is not yet clear whether the transition into these lineages occurs through an analogous bipotent progenitor.

### scRNA-seq analysis is consistent with a developmental hierarchy of progressive lineage restriction

To seek further evidence for progressive lineage restriction, and gain insight into the molecular basis for fate specification, we turned to single-cell (sc)RNA-seq analysis, focusing on E14 and E16 data reported by Hauser *et al*. ^29^ E14 represents an early progenitor phase that overlaps with our tracing data (Figure 1B), while E16 is the stage at which acinar, myoepithelial and ductal lineages are becoming specified. ^23^

**Figure 3:**
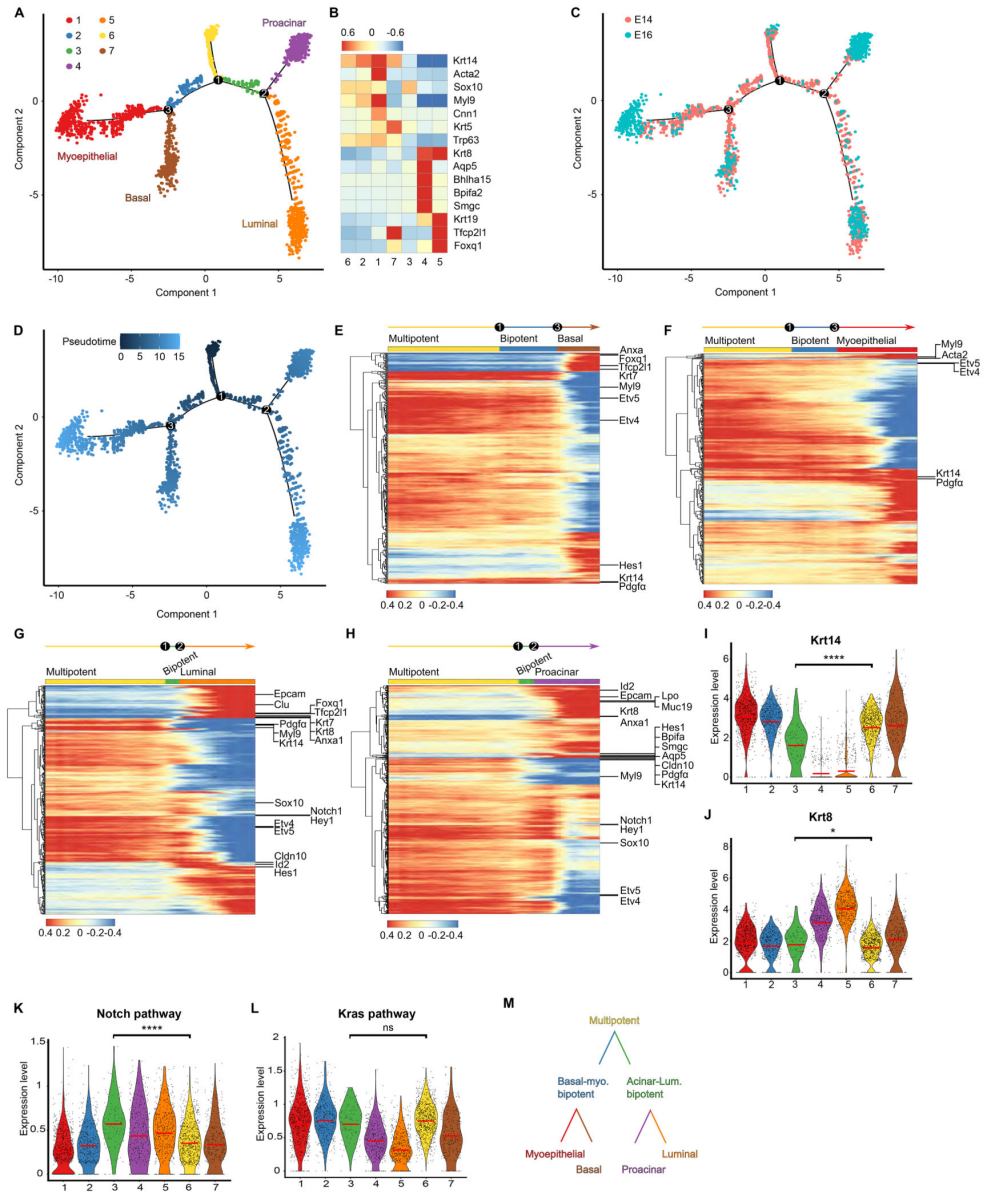
scRNA-seq analysis consistent with progressive lineage restriction. (A) Monocle map showing 3 branching points and 7 cell states inferred from pseudotime analysis of epithelial cells at E14 and E16. Black circles: branching points. Color legend: cell states. (B) Heatmap showing expression of representative marker genes for states in A. (C) Monocle map showing distribution of epithelial cells at E14 and E16. (D) Monocle map showing distribution of pseudotime with state 6 as starting point. Color bar denotes pseudotime. (E-H) Heatmaps showing changes in expression of differentially expressed genes along each trajectory from multipotent progenitor to the 4 lineages: basal duct (E), myoepithelial cell (F), luminal duct (G), proacinal cell (H). Color bar on top: cell state. Names of key genes denoted on right. See Table S5. (I-J) Violin plots representing expression of *Krt14* and *Krt8* per cell state in A. (K-L) Violin plots showing averaged expression of genes in Notch and Kras pathway per cell state in A. See Methods and Figure S5. (M) Schematic indicating lineage trajectories inferred by pseudotime.

We first integrated scRNA-seq data from E14 and E16, focusing on epithelial cells (Figure S5A-D and Table S4). Using pseudotime analysis (Supplementary Methods), we identified 7 states separated by 3 bifurcation points (Figure 3A,B): State 6 was sited at the apex of the first bifurcation (point 1) and consisted mainly of E14 cells that were annotated as endbud (Figure 3A-C and Figure S5D), showing little or no expression of differentiation markers (Figure 3B and Figure S5E-L). By contrast, cells positioned beyond bifurcation points 2 (states 4,5) and 3 (states 1,7) consisted of E14 and E16 cells (Figure 3C) that showed relatively high expression of differentiation markers of either the acinar, luminal, myoepithelial or basal lineage (Figure 3A,B and Figure S5E-H). Based on this analysis and the results of lineage tracing, we used state 6 as the entry point of a lineage hierarchy (Figure 3D): a multipotent state 6 branches into bipotent states 2 and 3 that produce, respectively, unipotent acinar (state 4) and luminal (state 5) progenitors, and unipotent basal (state 7) and myoepithelial (state 1) progenitors (Figure 3A,B).

We then questioned whether the two branches could be distinguished by marker gene expression. Examination of the transcriptional changes occurring within different trajectories showed that *Krt14* and *Krt8* have mutually exclusive expression (Figure 3E-J, Figure S5M,N and Table S5). Although *Krt14* remains elevated across the trajectories, producing myoepithelial and basal lineages, its expression level is reduced overall when multipotent cells enter the putative acinar-luminal bipotent state (state 3) and then diminishes as cells enter the acinar and luminal committed state (Figure 3I and Figure S5M). By contrast, *Krt8* is elevated in the acinar and luminal committed state, whereas it remains low in the multipotent and bipotent state and into the basal and myoepithelial committed states (Figure 3J and Figure S5N). Interestingly, genes associated with Notch signaling were more enriched in cells that follow the acinar and luminal trajectories, especially cells found in the acinar-luminal bipotent state, compared to the myoepithelial and basal trajectory (Figure 3K). On the other hand, genes associated with Ras signaling were highly enriched in endbud cells, including cells of the acinar-luminal bipotent state, and were particularly low in cells with luminal identity (Figure 3L).

Overall, consistent with the clonal analysis, the scRNA-seq data supported a hierarchical model of lineage segregation in which a multipotent progenitor, marked by expression of *Krt14*, gives rise to an intermediate bipotent acinar/luminal progenitor, marked by lower levels of *Krt14*, and a bipotent myoepithelial/basal progenitor, marked by high levels of *Krt14*. As cells of the acinar-luminal bipotent state transition towards the acinar and luminal differentiation state further reduce *Krt14* expression and increase *Krt8* (Figure 3M).

### Targeted lineage tracing supports existence of two populations of renewing bipotent tip progenitors

**Figure 4:**
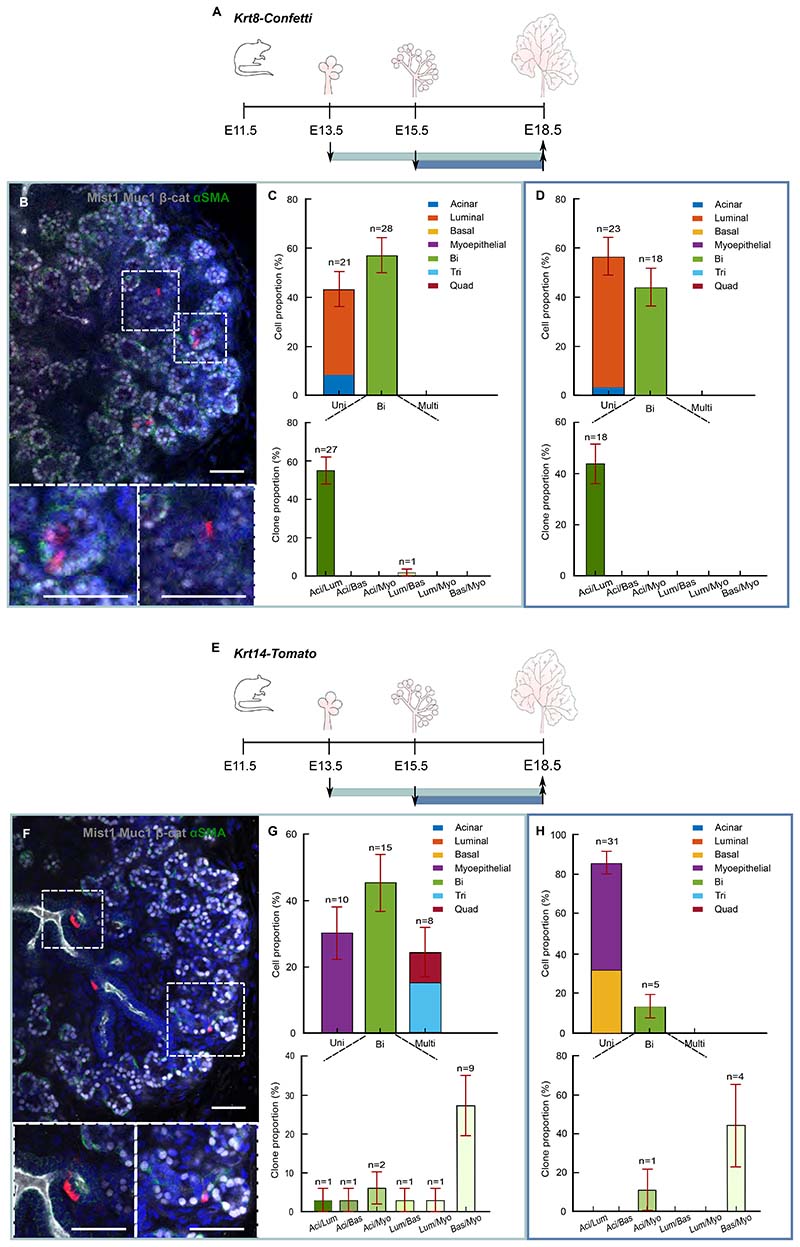
Targeted lineage tracing supports existence of two populations of renewing bipotent progenitors. (A) *Krt8-Tomato* mice were clonally induced at E13.5 (green line) or E15.5 (blue) and SMGs were collected at E18.5. (B) Bipotent clone from E13.5-E18.5 tracing (green bounding box). Magnified image of B showing labelled acinar cells (left box) and luminal cells (right box). Tissue stained as in Figure 1A. Scale bar: 50μm. (C) Proportion of unipotent, bipotent and multipotent clones (tri- and quad-potent) traced from E13.5-E18.5 ±SEP (green bounding box). n=49 clones from 9 mice, where 3 were pooled into 1 biological replicate. See Table S1. (D) Proportion of uni-, bi- and multipotent clones (tri- and quad-potent) from *Krt8-Confetti* mice traced from E15.5-E18.5 ±SEP (blue bounding box). n=41 clones from 9 mice, where 3 were pooled into 1 biological replicate. See Table S1. (E) *Krt14-Tomato* mice were clonally induced at E13.5 (green line) or E15.5 (blue) and SMGs collected at E18.5. (F) Bipotent clone from E13.5-E18.5 tracing (green bounding box). Magnified image of F showing labelled basal ductal cells (left box) and myoepithelial cell (right box). Tissue stained as in Figure 1A. Scale bar: 50μm. (G) Proportion of uni-, bi- and multipotent clones (tri- and quadripotent) from *Krt14-Tomato* mice traced from E13.5-E18.5 ±SEP (green bounding box). n=33 clones from 9 mice. See Table S1 and Methods. (H) Proportion of uni-, bi- and multipotent clones (tri- and quadripotent) from *Krt14-Tomato* mice traced from E15.5-E18.5 ±SEP (blue bounding box). n=36 clones from 9 mice. See Table S1, Figure S6, S7 and Methods.

To confirm the existence and identity of multi- and bipotent progenitors, we turned to targeted fate mapping using *Krt14creERT* to mark multipotent cells as well as cells that follow the myoepithelial and basal lineage, and *Krt8creERT* to mark cells that follow the acinar and luminal lineage (Figure 4). Immunostaining confirmed a mutually exclusive pattern of Krt14 and Krt8 protein expression, as indicated by scRNA-seq (Figure S6A). As previously described, ^21,25–27^ Krt14 expression was enriched in cells positioned at the outer layer of the endbud as well as ductal basal cells (Figure S6A). However, Krt8, which in adult SGs mark ductal cells ^30^, was found at the inner layers of the endbud and luminal ductal cells (Figure S6A). As with the *RosaCreERT* analysis, TAM was administered either at E13.5 or E15.5 and clones were traced to E18.5. Due to the low recombination efficiency of the confetti model, we used *Tomato* mice as a reporter line for *Krt14creERT* (*Krt14-Tomato*). To ensure that *Krt8creERT* labels exclusively the inner layer of the gland, we monitored the position of cells one day after TAM administration (Figure S6B-D). As expected from protein expression, recombined *Krt8*-positive cells were found exclusively at the inner layers.

Interestingly, consistent with a hierarchical model of lineage restriction, *Krt8creERT;Confetti* clones produced largely only acinar and luminal ductal cells, even from E13.5 induction, with only 1 basal cell out of 499 labelled cells and no myoepithelial cells (Figure 4A-D, Figure S6E-L and Figure S7A,B). Labelled acinar and luminal cells were observed either as part of the same (bipotent) clone or within lineage-restricted (unipotent) clones (Figure 4A-D). In contrast, *Krt14creERT;Tomato* clones induced at E13.5 gave rise to all 4 cell types including, in some cases, in the same (multipotent) clone (Figure 4E-G, Figure S6E and Figure S7C,D). However, from the E15.5 induction, the abundance of acinar cells was only 0.8±0.8% (±SEP) of the total output, compared to 95±1% (±SEP) and 24±3% (±SEP) from *RosaCre-Confetti* and *Krt8-Tomato*, respectively, while the majority of cells were of myoepithelial and basal ductal type (Figure 4E,H and Figure S6F). This is in line with previous tracing studies based on mosaic labelling, where *Krt14*+ cells induced at E16.5 lose their ability to produce acinar cells and give rise only to ductal and myoepithelial cells. ^12^ Despite the early induction of labelling (E13.5), clones produced from *Krt8* and *Krt14* were 5 times smaller overall than clones produced from *RosaCre-Confetti* (Figure S6G-L). To rationalize these differences, we noted that clonal labelling with *Krt14creER* and *Krt8creER* might favor labelling cells with higher marker expression which, as a result, may be both more fate restricted towards the myoepithelial and luminal ductal lineage, respectively, and less proliferative. Consistently, scRNA-seq showed that cells with higher levels of Krt14 (states 1,2,7) or Krt8 (states 4,5) have lower expression of G2M phase genes (Figure S5O,P). Consistent with psuedotime analysis, these results suggest that the final stages of lineage progression are marked by elevated expression of the respective lineage markers and a loss in proliferative activity. Note that clones produced from Krt8 and Krt14 were overall of similar size (11±3 (SEM) for Krt14 and 10±1 (SEM) for Krt8, p=0.9) (Figure S6G) showing that changes in potency are not influenced by proliferative potential.

Taken together, these results indicate that, at early stages of development, multipotent Krt14+ progenitors localize to the outer layer of endbuds, coexisting with bipotent Krt8+ progenitors. During development, Krt14+ progenitors gradually become restricted to myoepithelial and/or basal ductal fate. Whether the transition from multi- to bipotency, or from bi- to unipotency occurs in a manner that is temporally coordinated across an endbud, or whether multi-, bi- and unipotent progenitors can co-exist within the same endbud across serial rounds of branching is beyond the scope of the present study. However, our finding that some multi-, bi- and unipotent clones share the same CA (Table S2) suggests that such state transitions are unlikely to be synchronized across the endbud.

### Segregation of tip-localized bipotent progenitors is mediated by Notch signaling

Given the correlation of lineage restriction with position in the endbud, we hypothesized that progenitors at different locations might be exposed to differential signaling cues. As shown above, scRNA-seq analysis identifies Notch signaling as a pathway enriched at the inner *Krt8*+ epithelial core (Figure 3I-K). This pattern of expression was validated by immunostaining, which showed Hes1+ cells preferentially located at the inner layer (Figure S7E) and *Jag2*, a ligand for Notch, at the outer layer (Figure S7F).

**Figure 5:**
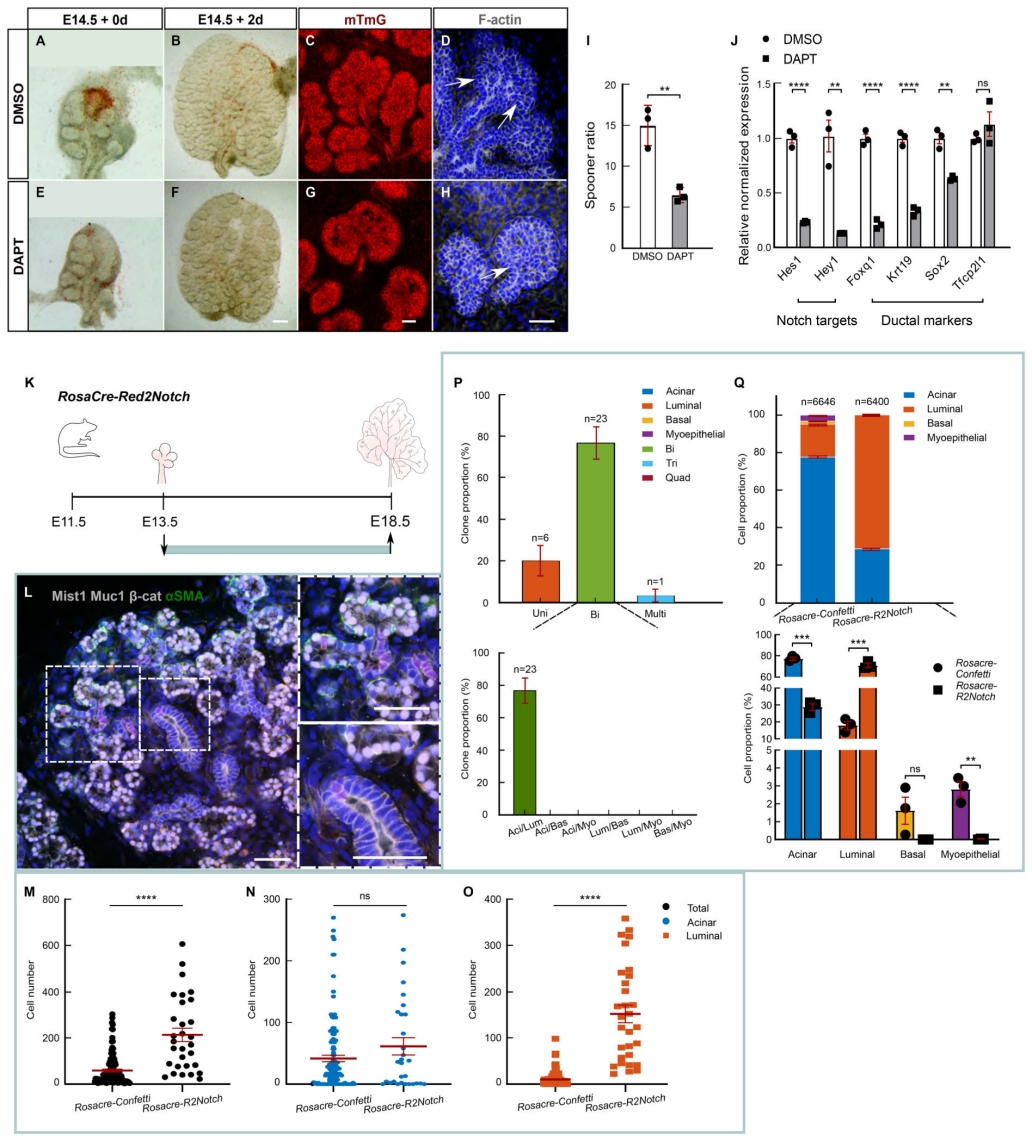
Segregation of tip-localized bipotent progenitors is mediated by Notch signalling. (A-H) Bright field images of embryonic SGs on day of dissection (E14.5) (A,E) and at 2 days after treatment (B,F) with DMSO (B) or Notch inhibitor DAPT (F). Scale bars: 200μm. C,G. Higher magnification images of SG explants in B and F at 2 days after treatment with DMSO (C) or DAPT (G). Red: mTmG. Scale bars: 50μm. D,H. Immunofluorescence staining of F-actin with Phalloidin (grey) on SG explants after 1 day of treatment with DMSO (D) or DAPT (H). Blue: DAPI. Scale bars: 50μm. (I) Spooner ratio (endbud number at 2 days after treatment/endbud number at day 0). n = 3 mice. (J) Expressional analysis of SMG explants treated for 1 day with DMSO (white) or DAPT (grey). Note that expression of *Tcfp2l1*, which acts upstream of Notch ^55^, remained unaffected. n = 3 mice. See Methods. (K) *RosaCreRed2Notch* mice were clonally induced at E13.5 and SMGs collected at E18.5 (green line). (L) Representative image of a red (Notch^icd^ active) clone at E18.5 (green bounding box). Magnified image of K showing an RFP subclone with exclusive luminal ductal contribution (top) or at an intra-lobular duct with luminal contribution (bottom). Tissue stained as in Figure 1A. Scale bars: 50μm. (M-O) Cell number (mean ±SEM) of all clones (M) and of acinar (N) and luminal (O) component of all clones in *RosaCre-Confetti* and *RosaCre-Red2Notch* at E18.5 (green bounding box). n = 112 clones in total for *RosaCre-Confetti* and 30 clones for *RosaCre-Notch*. See Methods. (P) Proportion ±SEP of uni-, bi- and multipotent clones (tri- and quadripotent) from *RosaCre-Red2Notch* mice traced from E13.5-E18.5 (green bounding box). n = 30 clones from 3 mice. See Table S1 and Methods. (Q) Proportion of labelled cell types from *RosaCre-Confetti* and *RosaCre-Red2Notch* at E18.5 (green bounding box). Above: Proportion ±SEP of all 3 biological replicates. n = cells. Below: average proportion ±SEM of cell types for each biological replicate. n = 3 mice. See Table S1, Figure S7 and Methods.

To investigate the functional role of Notch, we first treated E14.5 SG explants with N-[N-(3,5-Difluorophenacetyl)-L-alanyl]-S-phenylglycine t-butyl ester (DAPT), an inhibitor of Notch signaling. ^31^ Two-day treatment severely reduced branching as compared to controls, with explants displaying larger mesenchymal space, indicative of a reduced density of epithelial cells (Figure 5A-I). Closer examination revealed an enlargement of buds that lacked newly formed luminal cells, as outlined by F-actin deposition (Figure 5D,H arrows). ^27,32^ Expression analysis of duct-specific markers including *Krt19* and *Foxq1* confirmed this observation (Figure 5J).

We then questioned whether Notch could affect fate specification. To trace cells with perturbed Notch signaling, we used a modified version of the confetti line. ^33^ Upon induction, *Red2Notch* mice express randomly one of 4 fluorescent proteins (CFP, RFP, GFP and YFP) with the additional expression of *Notch1^icd^* in RFP+ cells. This allowed the simultaneous labelling of mutant and WT cells in the same tissue with non-red clones serving as an endogenous control. To label cells in an unbiased manner, we used *RosaCreERT* to induce expression of the *Red2Notch* construct at E13.5, and glands were collected at E18.5 (Figure 5K,L). Cells mutant for *Notch1^icd^* produced clones of larger sizes (Figure 5M), an increase due mainly due to the higher production of luminal cells (Figure 5N-Q). Moreover, myoepithelial cells in the RFP population accounted for only 0.01±0.01% (±SEP) of the total number of labelled cells compared to 3.0±0.2% (±SEP) in the *RosacreERT;Confetti* (Figure 5Q).

Interestingly all acinar cells produced with *Red2Notch* belonged to bipotent acinar/luminal or multipotent clones, and not unipotent clones (Figure 5P). Indeed, unipotent clones were only found for the luminal ductal lineage. This contrasts with *RosacreERT;Confetti*, where 24±4% (±SEP) of clones were of unipotent acinar type and 15±3% (±SEP) were of unipotent luminal type (Figure 1G). Together, these results favor a model in which the activation of Notch in unipotent acinar progenitors can redirect them towards luminal fate.

Finally, to confirm the role of Notch in the differential signaling of inner and outer layers, we traced the progenies of cells that were low in Notch using a conditionally activated dominant negative form of Mastermind like 1 line fused with GFP (*dnMaml1-GFP*) ^34^ (Figure S7G). Using the *Rosa* promoter for *CreERT* expression, mice were induced at mosaic density at E13.5 and tissue collected at E18.5. In this case, the luminal production was drastically reduced (Figure S7G) supporting the conclusion that luminal fate is determined by the level of Notch activation.

### Segregation of acinar and luminal ductal lineages is mediated by RAS signaling

Given that Notch promotes luminal over acinar fate, we investigated the pathways that promote acinar fate. As shown by scRNA-seq, Ras signaling is highly enriched in cells of the developing endbud, while remaining low in cells of the lumen (Figure 3L). This pattern of expression agrees with studies showing high activation of Erk signaling, one of the downstream components of the Ras pathway, required to support branching by maintaining endbud progenitors in an undifferentiated state. ^10,35–38^

**Figure 6:**
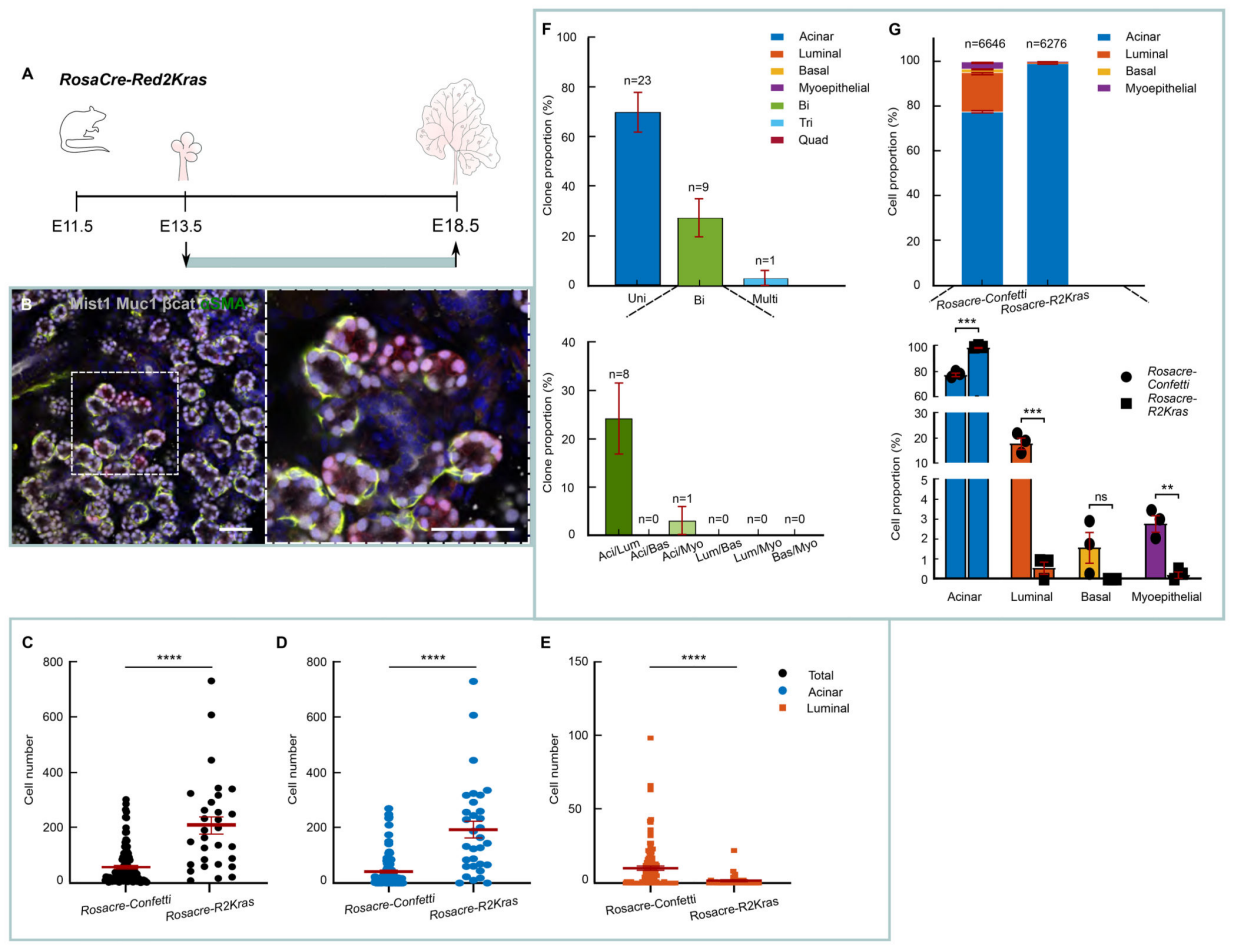
Segregation of acinar and luminal ductal lineages is mediated by RAS signalling. (A) *RosaCre-Red2Kras* mice were clonally induced at E13.5 and SMGs were collected at E18.5. (B) Representative image of RFP clone at E18.5 (green bounding box). Box: magnified image of B showing RFP subclone at terminal level with exclusive acinar contribution. Tissue stained as in Figure 1A. Scale bars: 50μm. See Figure S7. (C-E) Cell number (mean ±SEM) of all clones (C) and of acinar (D) and luminal (E) component of all clones in *RosaCre-Confetti* and *RosaCre-Red2Kras* at E18.5 (green bounding box). n = number of clones, 112 for *RosaCre-Confetti* and 33 for *RosaCre-Red2Kras* from 3 mice. See Methods. (F) Proportion ±SEP of uni-, bi- and multipotent clones (tri- and quadripotent) from *RosaCre-Red2Kras* mice traced from E13.5-E18.5 (green bounding box). n = 33 clones from 3 mice. See Table S1 and Methods. (G) Proportion of labelled cell types from *RosaCre-Red2Confetti* and *RosaCreRed2Kras* at E18.5 (green bounding box). Above: proportion ±SEP of all 3 biological replicates. n = cells. Below: Average proportion ±SEM of cell types for each biological replicate. n = 3 mice. See Table S1 and Methods.

To investigate the potential role of Ras signaling in cell fate, we made use of the *Red2Kras* line which, in common with *Red2Notch*, expresses randomly one of the 4 fluorescent proteins (CFP, RFP, GFP and YFP), with the RFP locus coupled in this case to the activation of *Kras^G12D^*. Once again, we made use of *RosaCreERT* to induce unbiased labelling (Figure 6A,B). Red cells mutant for *Kras^G12D^* produced overall clones of bigger sizes (Figure 6C). Strikingly, in this case, the increase was mainly due to the higher production of acinar cells (Figure 6D,E). Altogether, quantitative analysis of mutant clones showed a major differentiation bias towards acinar fate (Figure 6F,G). In contrast to *Red2Notch*, unipotent cells were only found for the acinar lineage (Figure 6F). Since, for WT, luminal cells are found in bi- and unipotent clones, this suggests that Kras activation can convert otherwise lineage-restricted (unipotent) ductal progenitors into bipotent progenitors that can also produce acinar cells (Figure 6F). In some cases, clonal activation of *Kras^G12D^* induced the formation of low intensity Mist1+ cell clusters (35%±0.5% (±SEP) of RFP labelled cells). These cells were excluded from our analysis since this phenotype was associated more with the oncogenic properties of *Kras^G12D^* ^39^ (Figure S7H,I and TableS1). Notably, these clusters had an adenoma-like morphology and they were easily distinguished from differentiated acini as the latter stained strongly for Mist1 and had larger cytoplasmic space (Figure S7J-M). As expected, RFP+ acini had robust nuclear pERK expression due to Kras^G12D^ induction and could express the acinar/intercalated marker Aqp5 (Figure S7L,M).

**Figure 7:**
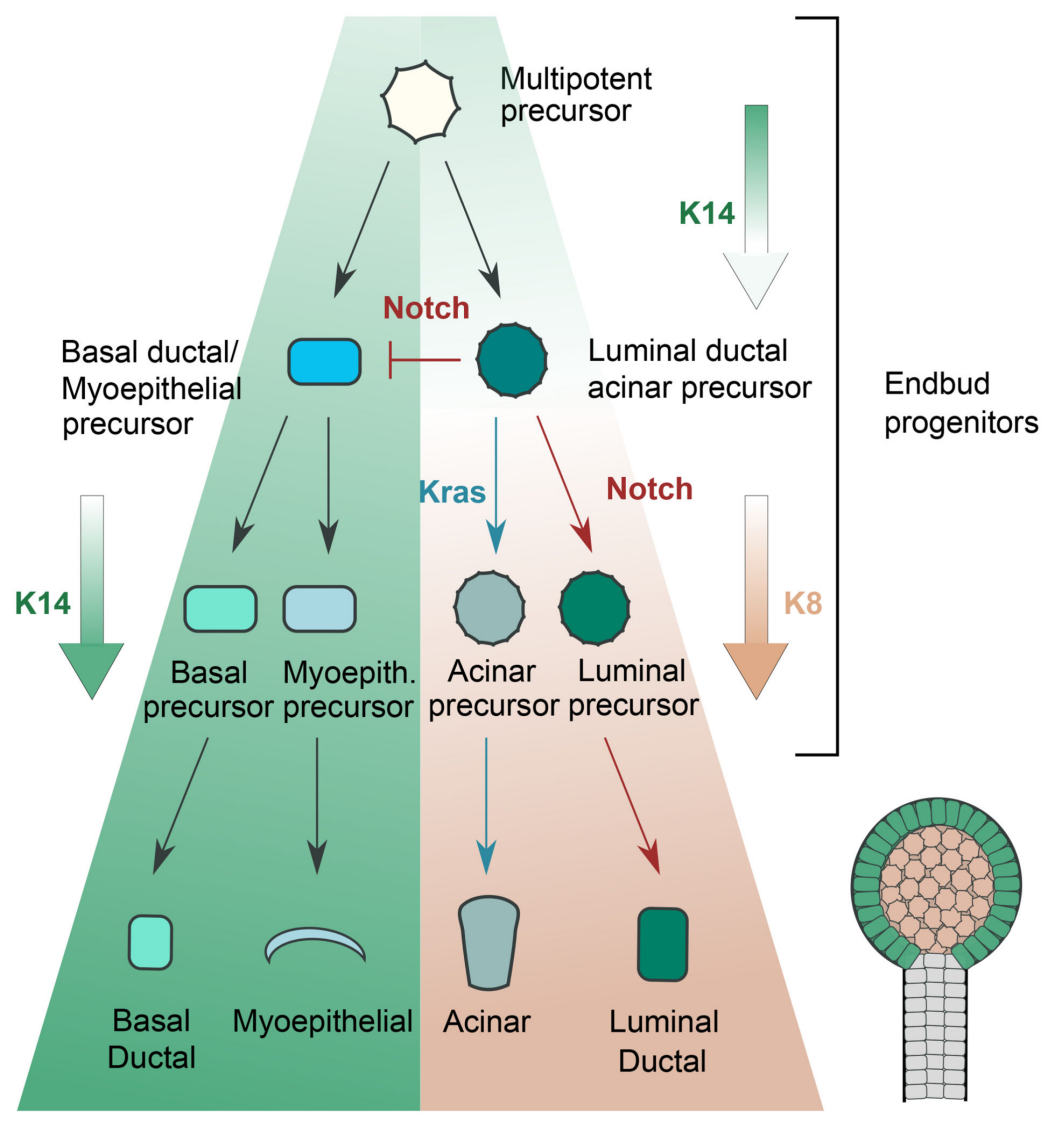
Hierarchical model of lineage segregation of endbud progenitors in SGs. Summary of pattern of lineage restriction and major pathways driving segregation.

Together, lineage tracing and perturbation analysis support a hierarchical organization of lineage segregation with Krt14+ multipotent progenitors giving rise to distinct bipotent progenitors marked by Krt14 or Krt8. Segregation of Krt14 and Krt8 bipotent populations involves Notch signaling, with sustained Notch1 activation favoring formation of luminal cells, while sustained Kras activation favors formation of acinar cells (Figure 7).

## Discussion

Previous studies have identified pathways that contribute to the development of the mouse SG epithelium. ^40,41^ However, the cellular events that drive branching and fate specification have remained in question. Here, we used clonal lineage tracing, molecular profiling and perturbation studies to define the lineage potential and pattern of fate specification during branching morphogenesis. Our results show that endbuds comprise a heterogeneous population of self-renewing progenitors that undergo a gradual process of lineage restriction, the timing of which occurs in a temporally overlapping manner.

### The timing of lineage specification

As SGs develop from the placode to the terminal bud stage, tip-localized progenitors gradually restrict their fate and commit to specific lineages. To address the pattern, timing, and stability of fate restriction, previous studies have focused on tracing strategies, targeting cells that are beginning to express differentiation markers such as the myoepithelial marker *αSma* ^11,12^, the acinar/intercalated marker *Aqp5* ^9,22^, and the ductal progenitor marker *Ascl3* ^42^. These population-level studies have suggested that, in most cases, lineage commitment is present at the time of marker expression. However, the timing of specification, and heterogeneity between individual progenitors, has remained in question. Using unbiased clonal lineage tracing, we found that lineage restriction can occur as early as E14.5, with 40% of clones already committed to either the ductal or acinar lineage.

This finding is resonant with tracing studies in the pancreas and mammary gland, which show that the majority of progenitors induced even at an early stage of development are unipotent. ^14–16,43,44^ Moreover, from the targeted tracing of *Krt14*+ progenitors, we found that specification of myoepithelial cells can initiate as early as E14.5, with some 27% of *Krt14+* progenitors showing unipotency at the E13.5 induction. This striking heterogeneity was hidden from previous populational-based *Krt14* tracing strategies. ^12^ It follows, therefore, that the timing of cell specification is heterogeneous and can precede the expression of cell-specific differentiation markers. Moreover, restriction into one of the 4 lineages does not interfere with the renewal potential of progenitors, allowing them to proliferate through serial rounds of endbud duplication.

### Lineage specification signals

The developing endbud is partitioned into an outer layer, consisting of (Krt14+) cells with high affinity to the basement membrane, and the inner core, with (Krt14-) cells having higher intercellular connections ^21,25–27^. The exchange of cells between these two compartments is low, with cells at the outer layer maintaining their identity even though they submerge temporarily into the inner layer to divide. ^21^ To what extent do these cellular movements affect cell fate choice?

Our unbiased lineage tracing revealed a progressive pattern of fate restriction, with multipotent progenitors transitioning into distinct populations of bipotent progenitors, committed either to the ductal basal/myoepithelial lineage or the ductal luminal/acinar lineages. To investigate whether this transition is linked to the establishment of layer identity, we turned to scRNA-seq analysis. Here, we identified discrete cell clusters, in which the identity of bipotent progenitors correlated with layer identity. To validate these findings, we used a targeted tracing strategy based on *Krt14creERT* (outer) and *Krt8creERT* (inner). The results supported a hierarchical model of lineage restriction, with a multipotent *Krt14*+/*Krt8*- progenitor giving rise to a *Krt14*+ bipotent progenitor restricted to the basal ductal-myoepithelial lineage and a *Krt8*+ bipotent progenitor restricted to the acinar-luminal ductal lineage. The emergence of myoepithelial and basal ductal cells from a common progenitor is consistent with previous scRNA-seq analysis at postnatal day 8 (P8) as well as population-level tracing studies, where cells expressing the myoepitehalial marker αSma at E16.5 collectively produced myoepithelial cells and a small proportion of basal ductal cells. ^11^ By inhibiting exchange of cells between layers during branching, outer layer cells may become biased towards the outer-located cell types (myoepithelial and basal ductal) and the inner cells towards to inner-located cell types (acinar and luminal).

Cells between the outer and inner layer were found to be exposed to different signaling molecules. In common with pubertal mammary glands, Notch signaling showed a preferential enrichment in the inner layers of the endbud and luminal ductal cells, with the outer layer and the basal ductal cells showing very low levels. ^45^ On the other hand, consistent with previous findings, Kras signaling was highly active at the developing endbud and low in luminal ductal cells. ^10,35–38^

How do Notch and Kras signaling affect cell fate specification? Numerous growth factors, as well as some transcription factors and extracellular matrix proteins, have been shown to regulate the formation of progenitors during branching. ^9,40,46^ Although informative, these studies are limited by the fact that the perturbation of signaling is applied uniformly, and therefore affecting the pattering of the whole epithelium. To circumvent this problem, we induced labelling and constitutive activation of Kras or Notch signaling at clonal induction to follow the progenies of cells with perturbed signaling when developing in a WT background. In common with postnatal mammary glands, we found that clonal activation of Notch promotes luminal fate. ^15,45^ In addition to Notch, Wnt, Egf, and Rock, RA, the Hippo pathway, and the parasympathetic ganglion have been shown to regulate the formation of ductal progenitors. ^32,37,38,47–52^ In future, it would therefore be interesting to investigate whether there is crosstalk between Notch and these pathways.

In contrast to Notch, clonal induction of Kras promotes acinar fate. In the developing SG, Kras is triggered by a variety of growth factors, the most essential of which are Fgfs, which have been shown previously to promote proacinar cell formation by a process that involves Sox10, Sox2 and cKit. ^13,48,50,53^ In addition, Etv1, a downstream target of Kras, ^54^ was identified as a putative acinar differentiation factor and could be involved in the mechanism of acinar specification through Kras. ^29^ Therefore, the balance between Notch and Kras may determine the decision to differentiate towards luminal or acinar fate, respectively, by a process that could involve other signaling pathways.

Interestingly this model of progressive lineage segregation during branching morphogenesis differs from adult, where each compartment is self-maintaining. ^9^ However, remnants of the embryonic program are still found at postnatal stages. For example, Kit+ progenitors located at the intercalated ducts of P2 mice can produce collectively both ductal and acinar cells. ^12^ In future, it will be important to investigate the programs that regulate the transition from embryonic to postnatal and then the adult stage to understand how adult homeostasis is achieved.

### Limitations of study

Due to the low induction efficiency of the *Krt14creER-Tomato* and *Krt8creER-Confetti* construct in developing SGs, and the need to ensure clonal density labelling, it was not possible to label *Krt14+* and *Krt8+* cells in a manner that is representative of tissue. Instead, the data suggest that cells that express higher levels of *Krt14* and *Krt8* are preferentially labelled. This resulted in clones of smaller sizes compared to the *RosacreERT* system (Figure S6G). However, we believe that this limitation does not impact on the results for clonal potency and the conclusions that follow. Indeed, when glands were induced non-clonally at E13.5 using *Krt8creERT-Tomato* *Krt8+*, cells retained their bias towards acinar (47.4±0.2% (±SEP)) and luminal (52.7±0.7% (±SEP)) fate, with no myoepithelial and basal cell contribution (n=3 mice, 264 cells quantified).

## Methods

### Resource availability

#### Lead contact

Further information and requests for resources and reagents should be directed to and will be fulfilled by the lead contact, Benjamin D. Simons (bds10@cam.ac.uk).

#### Materials availability

This study did not generate new unique reagents.

## Experimental model and subject detail

### Mouse models

All experiments were performed according to the Home Office regulations and approved by the University of Cambridge Animal Welfare and Ethical Review Body. Mice were kept in a pathogen-free facility in individually ventilated cage under a 12-hour light and dark cycle. Mice had an unlimited 24h-access to food and water. Cages were changed routinely and a maximum of 5 adult mice were housed in the same cage. The health of the mice was monitored daily and only healthy mice were used for this study. The *Rosa26-CreERT2* (RRID:IMSR_JAX:008463) ^56^, *Rosa26-Confetti* (RRID:IMSR_JAX:013731) ^20,57^, *Krt14-creERT2* (RRID:IMSR_JAX:005107) ^58^, *Krt8-creERT2* (RRID:IMSR_JAX:017947) ^59^, *Rosa26-Red2Kras* ^33^, *Rosa26-Red2Notch* ^33^, *Rosa26-TdTomato* (RRID:IMSR_JAX:007914) ^60^ and *Rosa26-dnMaml1* (RRID:IMSR_JAX:032613) ^34^ mice have been previously described. All mice were maintained on a C57BL/6 background to minimize variation in the gestation length. Since there is no sexual dimorphism at the embryonic stage, we did not distinguish between males and females ^66^. The embryonic stages used were of E14.5, E16.5 and E18.5. Adult females of 6-20 weeks of age and males of 6-32 weeks of age were used for breeding.

## Method details

### Lineage tracing *in vivo*

Tamoxifen (Sigma) was prepared in a stock concentration of 20mg/ml and stored at -80°C. For clonal induction, tamoxifen was given by oral gavage at the following concentrations: For *Rosa26-CreERT2;Rosa26-Confetti*, *Rosa26-CreERT2;Rosa26-Red2Kras*, and *Rosa26-CreERT2; Rosa26-Red2Notch* mice were induced with 0.05mg/body weight. *Rosa26-CreERT2;Rosa26-dnMaml1* mice were induced with 0.025mg/body weight. *Krt8-CreERT2;Rosa26-Confetti* mice were induced with 0.35mg/body weight at E13.5 and with 0.2mg/body weight at E15.5. *Krt14-creERT2;Rosa26-Tomato* were induced with 0.35mg/body weight and with 0.3mg/body weight at E15.5.

### Tissue preparation

Submandibular glands were dissected from timed mating, pregnant females. Day 0 was the day of the vaginal plug. To maintain the tissue architecture of the capsule, submandibular glands were dissected with the sublingual glands, as previously described ^6^. Salivary glands where then used either for explant culture or for fixation. Salivary glands of E14.5 to E15.5 were fixed for 30 min in 4% Paraformaldehyde (PFA) rocking at room temperature while salivary glands of E16.5 to E18.5 were fixed for 1 hour at the same conditions. Excess salivary gland tissue was stored in 30% sucrose at -20°C.

### Explant cultures

Salivary gland explants were cultured as previously described ^67,68^. Briefly, salivary glands were dissected at E14.5 and cultured on a 0.4μm pore filter (Falcon) floating on Advanced Dulbecco’s Modified Eagle Medium F12 (Advanced DMEM/F-12) (Gibco®) supplemented with 1% GlutamaxTM (Gibco®), 1% penicillin-streptomycin (Sigma-Aldrich), 150 μg/ml ascorbic acid and 50 μg/ml transferrin. Salivary gland explants were incubated at 37°C with 5% CO2. For Notch inhibition E14.5 salivary gland explants were treated for either 1 day or 2 days with either 20μM DAPT (Sigma) or DMSO as a control ^69^.

### Immunofluorescence of whole mount salivary glands

Rocking was used for each incubation step. Whole mount salivary glands were first permeabilized in 0.5% Triton 100x in PBS for 4 h and then blocked for 1 h in 2% donkey serum and 0.5% Triton-100X diluted in PBS (blocking solution). Salivary glands were incubated with the primary antibodies diluted in the blocking solution at the optimal concentration. The incubation time varied depending on the developmental stage. E14.5 to E15.5 glands were incubated for 4 days at 4°C, while E16.5 to E18.5 were incubated for 7 days at 4°C. Salivary glands were then washed 6 times for 1h in 0.5% Triton-100X PBS at room temperature and incubated with the secondary antibody (if required) diluted 1:500 in blocking solution for either 4 days (E14.5 to E15.5) or 7 days (E16.5 to E18.5) at 4°C. Following the incubation, the tissue was washed 6 times for 1h in 0.5% Triton-100X PBS at room temperature and mounted on 22 X 32 mm coverslips with a 0.25 mm i-spacer (Sunjin Lab) in RapiClear 1.52 (SunJin Lab). The mounted tissue was incubated overnight at 4°C to allow clearing of the samples and then imaged on the confocal microscope. The primary and secondary antibodies used were the following: anti-Mist1 (Abcam, ab187978), anti-Muc1 (Abcam, ab15481), anti-β-catenin (L54E2)-647 (Cell Signaling, 4627S), anti-α-Smooth Muscle-FITC (Merck, F3777), Alexa Fluor-647 Donkey Anti-Rabbit (Thermo Fisher Scientific, A31573).

### Imaging and Image analysis

Images of the whole salivary glands were obtained using the Leica SP8 X White Light laser confocal microscope. For 3D ductal reconstruction and clone mapping, individual images were taken with a 20x oil-immersed objective and were then stitched together using the Leica LASX software. The stitched image was analyzed using imageJ to determine the position and the cell identity of clones ^65^. A purpose-built graphical user interface (GUI) developed in Matlab R2020a (Natick, Massachusetts: The MathWorks Inc) was used for the manual ductal reconstruction, clone grouping and clone assignment.

### Cell type assignment

The cell type identity of the clones was assessed based on the epithelial marker β-catenin, acinar marker Mist1, luminal ductal marker Muc1 and myoepithelial marker αSma (Figure 1A). In addition to outlining the epithelium, staining with β-catenin allowed for easier characterization of the tissue architecture and aided to distinguish basal cells from luminal cells. To allow the visualization of all the four colors of the Confetti, AlexaFluore-647 was used to stain for Mist1, Muc1 and β-catenin due to the differential cellular localization of the proteins (nuclear, luminal and plasma membrane respectively). αSma conjugated with FITC was used to stain myoepithelial cells. The cytoplasmic location of αSma allowed to distinguish between the myoepithelial cells and the nuclear GFP positive cells of the confetti construct.

### Transcription analysis

Expression levels of mRNA was analyzed using quantitative PCR (qPCR). The RNA of the salivary glands was extracted from the salivary gland explants using the RNAeasy micro kit (Qiagen) with DNAse treatment according to the manufacturer’s instructions. Three salivary gland explants were pooled for each of the three biological replicates. 0.7 μg of the extracted RNA was used for cDNA synthesis with the GoTaq G2 Flexi DNA Polymerase (Promega). *Gapdh* was used as an endogenous control for normalization.

## Quantification and statistical analysis

For Figure 1G, n=112 clones from 3 mice. Top: Tripotent clones were mainly composed of acinar, luminal and myoepithelial cells (12.5±3.1% of all clones), then acinar, luminal and basal (2.7±1.5%), acinar, basal and myoepithelial (1.8±1.3%), and luminal, basal and myoepithelial (0.89±0.88%).

For Figure 1H, n=77 clones from 3 mice Tripotent clones were composed of acinar, luminal and myoepithelial cells (1.3±1.3% of all clones) and acinar, basal and myoepithelial (1.3±1.3%).

For Figure 2B and C, statistical analysis was performed using two sample Kolmogorov-Smirnov test. n=112 clones from 3 mice.

For Figure 3I-L, Wilcoxon test used and statistical significance denoted as follows: ns: non-significant, *: P < 0.05, ****: P ≤ 0.0001.

For Figure 4G, n=33 clones from 9 mice, where 3 were pooled into 1 biological replicate. Tripotent clones were composed of acinar, basal and myoepithelial cells (12.1±5.7% of all clones) of luminal, basal, myoepithelial (3.0±3.0%).

For Figure 4H n=36 clones from 9 mice, where 3 were pooled into 1 biological replicate.

For Figure 5I and J, statistical analysis was performed with unpaired t-test. Ns: non-significant, **: P ≤ 0.01, ***: P ≤ 0.001. n = 3 mice.

For Figure 5M and O, statistical analysis was performed using Mann Whitney test. Ns: non-significant, ****: P ≤ 0.0001. n = 112 clones in total for *RosaCre-Confetti* and 30 clones for *RosaCre-Notch*.

For Figure 5P, n = 30 clones from 3 mice. Unipotent clones contained only luminal ductal cells. Tripotent clones were composed of acinar, luminal, and myoepithelial cells (3.3±3.3% of all clones). Quadripotent clones were absent.

For Figure 5Q, statistical analysis was performed with unpaired t-test. Ns: non-significant, **: P ≤ 0.01, ***: P ≤ 0.001.

For Figure 6C-E, statistical analysis was performed using Mann Whitney test. ****: P ≤ 0.0001. The total number of clones was 112 for *RosaCre-Confetti* and 33 for *RosaCre-Red2Kras*.

For Figure 6F, n = 33 clones from 3 mice. Unipotent clones consisted exclusively of acinar cells. Tripotent clones were comprised of acinar, luminal, and myoepithelial cells only (3.0±3.0% of all clones). Quadripotent clones were absent.

For Figure 6G, statistical analysis was performed with unpaired t-test. Ns: non-significant, **: P ≤ 0.01, ***: P ≤ 0.001.

### Ductal network segmentation and clonal mapping

The three-dimensional ductal network of the first lobe was segmented from confocal z-stacks by manually tracing the lumen of the ducts, from the main duct of the lobe to the endbuds. Labelled cells where then manually identified and marked their spatial coordinates using ImageJ (Figure 1D-F, Movie S1, Movie S2 and Figure S2). Each labelled cell was then mapped onto the reconstructed ductal network by manually assigning the position of cells to the nearest duct using a purpose-built GUI in Matlab (Figure S2A-B). Although efforts were made to automate these processes computationally, the ductal density and thinning of the ductal lumen while approaching the endbuds, prevented a reliable segmentation and classification. As a result, all segmentations were performed and verified manually.

### Clonal grouping and filtering

Once all labelled cells were assigned to the segmented ductal network, groups of cells contained within the same subtree and expressing the same fluorophore color were manually grouped into putative clones. Altogether, 27% of renewing clones were found to be RFP+, 29% YFP+, 27% CFP+ and 8% GFP+. By using a low dose of Tamoxifen, we sought to mitigate the effects of clone merger events where clones of a common color are induced in the same endbud. Based on the relative incidence of clones having a different color and belonging to the same subtree, we estimated that the frequency of clone merger events to be less than 5%. Formally, if at the time of induction *N* endbuds experience a total of *M* labelling events in a confetti color *c*, the probability that an endbud is labelled by that color is equal to *P_c_* = 1 − (1 − 1/*N*)^*M*^ ≈ 1 − *e*^−*M*/*N*^ = 1 − *e*^−*p*^, where *p* = *M*/*N* is the average labelling probability. The probability that a labelled endbud of a given color is the product of a merger event due to the induction of two or more like-color cells in the same endbud is then given by *P*_Merger_ = 1 − *P*_1_/*P_c_* ≈ 1 − *p*/(*e^p^* − 1), where *P*_1_ = (*M*/*N*) (1−1/*N*)^*M*−1^ ≈ *pe*^−*p*^ is the probability that an endbud has acquired precisely one labelling event in that color. Therefore, for *p* ≪ 1, *P*_Merger_ ≈ *p*/2. The probability that an endbud is labelled any number of times in one and only one of the 4 confetti colors is given by *Q*_1_ = 4*P_c_*(1 − *P_c_*) ^3^. Similarly, the probability that an endbud is labelled in any combination of any of the four confetti colors is given by *Q*_∗_ = 1 − (1 − *P_c_*) ^4^. Therefore, the probability that an endbud is co-labelled by two or more confetti colors is given by *C* = 1 − *Q*_1_/*Q*_∗_ ≈ 3*p*/2 = 3 × *P*_Merger_. Experimentally, from the E13.5 induction time point, we find a total of 112 common ancestor endbuds of which 10 are co-labelled by cells of different colors so that *C* = 10/122 ≈ 0.09. This implies a merger probability of around *P*_Merger_ = *C*/3 = 0.03 or 3%. This estimate compared favorably with the results of stochastic simulation based on the measured ductal network topology (see below). Note that, in reality, the labelling efficiency is not equivalent between confetti colors, but clones (per gland) are found in the proportion: 10±2, 11±3, 3±1 and 13±2 (mean ± SD for *n* = 3 mice) for CFP, YFP, GFP and RFP colored clones respectively. This variability will adjust the estimate of the true merger frequencies but only to a small degree.

For the statistical analyses presented in this work, clones that were entirely contained within a single ductal segment of the network (termed “non-renewing”) were removed, as it was likely that these clones were derived from cells that had already committed to differentiation at the time of induction, i.e., they did not belong to the ensemble of progenitors that maintained renewal potential during rounds of consecutive branching. In rare cases (accounting for 4% of the total acinar cell number at E18.5), we identified individual acinar cells located within the luminal compartment of ducts up to four branching generations away from terminal acini (Figure S3C). These duct-associated acinar cells could be identified as early as E16.5 based on elevated Mist1 staining (Figure S3D). However, we note that duct-associated acinar cells have not been observed at the adult stage.

### Common ancestor and subtrees

Any given clone could span multiple ducts and/or endbuds of the branching tree. However, as all cells in a clone originate from a single labelled cell at the time of induction, such clones must be associated with an induced cell with renewal capacity. Here, by renewal, we mean the ability to duplicate and segregate during the process of endbud bifurcation. Moreover, at the time of induction, the induced cell must have been located on a tip that, together with its neighbors, must have had the potential to generate all the ducts of the resulting subtree that host the clone. We therefore reasoned that the likely location for the initially labelled cell of a renewing clone was associated with the duct corresponding to the last common ancestor of the subtree that contains all cells of the clone (Figure 2A). Although this assignment may be imprecise, it does provide a rigorous bound on the branch level of the labelled cell at the time of induction.

Operationally, the common ancestor was found by locating the duct at the lowest level of the tree from which all labelled duct and tips could be reached (Figure 2A). The subtree populated by a clone corresponded to the entire subtree that derives from the common ancestor of the clone; this might include ducts and tips that contain no labelled cells. Note that, to estimate the average number of renewing acinar tip progenitors, the minority of labelled acinar cells located within ducts (i.e., not contained within an acinus) were excluded from the analysis as they do not contribute to the formation of an acinus.

### Modelling the clonal dynamics of the branching tree

To build a null model of the clonal dynamics, we performed stochastic simulations of the dynamics of renewing tip-progenitors on the branching trees using the experimentally determined topology. More precisely, we simulated the dynamics of clones by placing a single (tip) cell in a branching tree, where the level of the starting node was an input parameter. At every time step, the cell was allowed to move one level up in the tree, choosing one of the adjacent nodes at random. In this process, at each round of duct bifurcation, the cell number was doubled, and a “unit” of duct density was assigned to the duct traversed. The process was iterated until all tip cells reached endpoints of the branching tree. From this data, we could obtain the predicted distribution of cells for a given clone on one particular subtree. To generate statistics, we simulated 1000 clones on each of the 3 experimentally measured networks (Figure S2K-M) and used this data to estimate the chance of clone merger, the predicted clone size distribution and the subclone density as a function of branch level.

### Fidelity of the clone assignment using stochastic simulation

Using stochastic simulations of the clonal dynamic on the measured branched networks we were able to assess numerically the likelihood of clone merger events, i.e., the chance that two or more clones of the same color populate a common duct. For this, we measured the average induction rate in the E13.5-E18.5 samples, which resulted in an average of 10 clones induced per color per experiment. Then, by simulating the simultaneous evolution of 10 clones in the empirical trees, all starting from level 7 in the tree, and performing 100 realizations of this numerical experiment, we estimated the fraction of clone merger events at 5% (consistent with the analytical estimate above). All clones were initialized at level 7 as this is the average position of the common ancestor in all three samples induced at E13.5 and measured at E18.5. It should be noted that, when clones are all initialized at levels 5 or lower, the likelihood of merger rises to 10% and above. Conversely, if initialized at levels 8 or 9, the chance of merger is reduced to 3% and 1% respectively, due to the increased number of nodes available to choose from.

For samples induced at E15.5 and measured at E18.5, we did not reconstruct the minimal ductal subtree containing the entire the clone. Thus, we could not access the level of the common ancestors of clones. However, based on estimations of the branching rate of endbuds of about once per day (Bordeu et al, unpublished), we expect merger events to still be present in 1-3% of the clones, based on an average common ancestor between levels 8-9 (1-2 levels higher than the position of the average common ancestor of clones induced at E13.5).

### Estimation of the number of progenitors

Once again, following ^16^, we reasoned that, after induction, a renewing progenitor cell would on average contribute a representative fraction of labelled cells to the resulting subtree. For example, if a unipotent acinar clone gives rise on average to, say, 2% of acinar cells in the subtree derived from the common ancestor, one might estimate that, at the time of labelling, the endbud contained some 100/2=50 unipotent renewing acinar progenitors that together give rise to all acinar cells on the subtree. However, since acinar-producing multipotent, bipotent and unipotent progenitors may coexist in the same endbud, further care must be taken to accommodate the respective contributions of progenitor subtypes. To account for this heterogeneity, we posited that the relative frequency of clone types was broadly representative of the relative proportion of progenitor types in endbuds at the time of labelling. Note that, in this case, the positional bias of induced cells may compromise the precise quantitative values for progenitor cell number. However our aim here was to obtain a broad estimate, questioning whether only a handful of cells in the endbud maintain renewal potential, as found in the mouse pancreas, or whether it is the majority, as observed in the mammary gland. Based on this reasoning, we used the average fractional contribution of progenitor types to subtrees to estimate their abundance (Figure 2H, Figure S4E, Table S3).

For a given cell compartment *c* (acinar, luminal, basal or myoepithelial), we define *f_u_*, *f_b_*, *f_m_* as the fraction of unipotent, bipotent and multipotent progenitors contributing to that compartment, respectively, with *f_u_* + *f_b_* + *f_m_* = 1. Then, defining *c_u_*, *c_b_* and *c_m_* as the relative contribution of each clone type (uni-, bi- or multipotent) to the corresponding subtree, the total number of renewing tip progenitors for compartment *c* can be estimated as *N_c_* = 100/(*f_u_c_u_* + *f_b_c_b_* + *f_m_c_m_*. From this result, the fraction of renewing tip progenitors corresponding to unipotent, bipotent and multipotent cells that contribute to the compartment in consideration are then given by *f_u_N_c_*, *f_b_N_c_* and *f_m_N_c_*, respectively. To calculate the relative contributions *c_u_*, *c_b_* and *c_m_*, we considered the subtree formed from the common ancestor of the clone in consideration and estimated the number of acinar, ductal, and myoepithelial cells in the subtree. Based on average cell counts, we estimated that a single acinus could host around 63 (with 95% confidence interval between 48 and 79) acinar and 6 (with 95% confidence interval between 5 and 7) myoepithelial cells on average, and that the linear density of luminal and basal ductal cells in ducts was about 0.29 ± 0.02 (SEM) and 0.25 ± 0.04 (SEM) cells/micron, respectively. This allowed us to estimate the total number of cells of each compartment in the subtree being considered. The ratio of the total number of cells of a given compartment in a clone and the total number of cells in the corresponding subtree, gave the relative contribution of a clone to each compartment in the subtree, whose average among all clones corresponded to the contributions of interest. Note that the estimate of *N_c_* assumes that the labelling is approximately representative of the tissue and endbud composition.

For example, for the acinar compartment, from the E13.5 induction time (sample 1), we found that 26 out of 54 clones in the sample contributed to the acinar pool, of which a fraction *f_u_* = 11/26 were unipotent, *f_b_* = 12/54 bipotent, and *f_m_* = 3/26 multipotent. A unipotent acinar clones gave rise, on average, to *c_u_* = 0.018 (or 1.8%) of all acinar cells in its corresponding subtree. Similarly, acinar-containing bipotent and multipotent clones gave rise, on average, to *c_b_* = 0.0056 (or 0.56%) and *c_m_* = 0.0058 (or 0.58%) of all acinar cells in their corresponding subtrees. From this result, it followed that the total number of renewing progenitors contributing to the acinar compartment was approximately *N_a_* = 94 cells (Figure 2I, Figure S4E and Table S3), of which 40 corresponded to acinar unipotent progenitors, while 43 to bipotent and 11 to multipotent acinar producing progenitors. By considering the estimates from the n=3 repeats, we found an average number of renewing progenitors in the early endbuds of *N_a_* = 88 ± 14 (mean±SD) for the acinar, *N_l_* = 66 ± 15 for the ductal luminal, *N_b_* = 138 ± 55 for the ductal basal, and *N_m_* = 80 ± 59 for the myoepithelial compartments. Considering the uncertainties, we note that all these values are within the estimated size of an endbuds at E14.5 of around 117 cells.

### Reanalysis of scRNA-seq data

#### Reintroducing embryonic single-cell profiling data

We obtained and reanalyzed scRNA-seq data of mouse salivary gland development from a previous study ^29^ deposited in the Gene Expression Omnibus (GEO) GSE150327. Among the 6 time points reported in this study, we made use of only two samples from E14 and E16, the first with 3,050 cells and the second with 899 cells. We selected E14 and E16 since these time points align well the timing of lineage restriction from the multipotent progenitor population. Given the emphasis of our study, we made use of the cell type annotation reported in the original paper ^29^ and focused only on epithelial cells, filtering out all cells other than endbud, myoepithelial, basal duct, and Krt19+ duct cells.

#### Data processing

Unique Molecular Identifiers (UMIs) were normalized by a deconvolution method using R package scran (v1.12.1) ^70^. To remove potential bias due to the cell cycle signature, we performed cell cycle regression analysis. Specifically, we calculated G1, S, and G2/M scores using cyclone function of scran and used the values to correct the normalized data by regressing out cell cycle using removeBatchEffect function of limma (v3.38.3) ^71^. PCA combined with technical noise modelling was applied to the corrected expression data for dimension reduction, which was implemented by denoise_PCA function of scran. The batch effect between different stages was then removed by using RunHarmony function of harmony (v1.0) with default parameters ^72^. The batch-corrected data was used for non-linear dimension reduction based on t-distributed stochastic neighbor embedding (TSNE) implemented in runTSNE function (default parameter used) of R package scater (v1.10.1) ^73^.

#### Cell type annotation and calculation of marker genes

We then performed Louvain clustering (k-nearest neighbor = 7) for the data without cell cycle effect, which resulted in 12 clusters. The 12 clusters were then classified into the following 6 cell states based on known marker genes shown in Figure S5C: endbud, myoepithelial, basal duct, proacinar, and luminal duct state, as well as cells undergoing epithelial-to-mesenchymal transition (EMT). The EMT cells were filtered out for further analysis. Then, we identified highly expressed (a.k.a. marker) genes satisfying a given criterion (i.e., FDR < 0.01, log2(fold-change) > 0.445) for each cell state compared to others at E16 using findMarkers function of scran with default parameters, as cells at E14 are not differentiated enough to identify the marker genes (Table S4).

#### Pseudotime analysis

We then aimed to infer a potential lineage relationship between the putative multipotent progenitor population and bipotent and unipotent progenitor lineages. To this end, we performed pseudotime analysis using the R package monocle (v2.18.0) for the epithelial cells at E14 and E16 ^74^. First, based on the marker genes above, dimension reduction was carried out by the DDRTree algorithm implemented in monocle. Next, the cells were ordered using orderCells function of monocle with default parameters to calculate pseudotime along lineage specification. As a result, 3 bifurcation points and 7 cell states were identified (Figure 3A). Based on the cell states, marker genes were calculated using the findMarkers function (FDR < 0.01 and log2(fold-change) > 0.5) with the batch effect from the different stages blocked. We then displayed the expression profiles for the marker genes along the pseudotime for each of four trajectories such as myoepithelial, basal duct, proacinar and luminal duct lineage (Figure 3E-H, Table S5). For each gene, auto-scaled gene expression was plotted using a rolling mean along its trajectory with a window size of 10% of cells. On the other hand, based on experimental clues, the averaged expression of downstream genes of Kras and Notch pathways were mapped along the pseudotime trajectories to infer the pathway activity along the fate specification. The downstream genes used for Notch were: *Hes1*, *Hey1*, *Notch1*, *Lfng*, *Nrap*, *Heyl* and *Maml1*. The downstream genes for Kras were *Fgfr1*, *Fgfr2*, *Spry1*, *Spry2*, *Etv1*, *Etv4* and *Etv5*.

The results generated here by pseudotime do not agree fully with the hierarchical organization proposed by Hauser et al., where differentiation into basal, myoepithelial and acinar lineages were thought to progress through luminal ductal progenitors. In this context, we would note that, in contrast to Hauser et al., we placed emphasis on data from the early embryonic stages and did not include data from postnatal and adult stages, which were beyond the scope of our tracing studies. It may be that this focus provides a finer resolution of the pattern of lineage restriction that becomes compromised when data is integrated across the broad range of developmental times.

## Supplementary Material

movie S1

movie S2

Tables S1

Tables S4

Tables S5

Supplemental Tables and Figures

## Figures and Tables

**Table:** Key resources table

REAGENT or RESOURCE	SOURCE	IDENTIFIER
Antibodies		
anti-Misti	Abcam	Cat# ab187978, RRID:AB_2924393
anti-Muc1	Abcam	Cat# ab15481, RRID:AB_301891
anti-β-catenin (L54E2)-647	Cell Signaling	Cat# 4627S, RRID:AB_2924879
anti-α-Smooth Muscle-FITC	Merck	Cat# F3777, RRID:AB_476977
Alexa Fluor-647 Donkey Anti-Rabbit	Thermo Fisher Scientific	Cat# A31573, RRID:AB_2536183
Chemicals, peptides, and recombinant proteins		
RapiClear 1.52	SunJin Lab	RC152002
DAPT	Sigma	D5942
Tamoxifen	Sigma	T5648
Deposited data		
Script for scRNA-seq, network reconstruction and clone mapping	This study	DOI: 10.5281/zenodo.7448197
scRNA-seq data for embryonic salivary glands	scRNA-seq generated by Hauser et. ^29^	
Experimental models: Organisms/strains		
Mouse: *Rosa26-CreERT2*	Ventura et al. ^56^	RRID:IMSR_JAX:00 8463
Mouse: *Rosa26-Confetti*	Livet et al. ^57^	RRID:IMSR_JAX:01 3731
Mouse: *Krt14-creERT2*	Vasioukhin et al. ^58^	RRID:IMSR_JAX:00 5107
Mouse: *Krt8-creERT2*	Van Keymeulen et al.^59^	RRID:IMSR_JAX:01 7947
Mouse: *Rosa26-Red2Kras*	Yum et al. ^33^	
Mouse: *Rosa26-Red2Notch*	Yum et al. ^33^	
Mouse: *Rosa26-TdTomato*	Madisen et al. ^60^	RRID:IMSR_JAX:00 7914
Mouse: *Rosa26-dnMaml1*	Tu et al. ^34^	RRID:IMSR_JAX:03 2613
Oligonucleotides		
*Hes1:* 5’-ACACCGGACAAACCAAAGAC; 5’- AATGCCGGGAGCTATCTTTC	Briot et al. ^61^	N.A.
*Hey1:* 5’- CACCTGAAAATGCTGCACAC, 5’- ATGCTCAGATAACGGGCAAC	Odelin et al. ^62^	N.A.
*Foxq1*: 5’-GCCTATTGAGTCTTAACCCTCC; 5’- GAGTGCGTTGGGATGAGAAT	This study	N.A.
*Krt19:* 5’-TGAAGATCCGCGACTGGTAC; 5’- GGCGAGCATT GT CAAT CT GT	This study	N.A.
*Sox2:* 5’-GCGGAGTGGAAACTTTTGTCC; 5’- GGGAAGCGTGTACTTATCCTTCT	Xiao et al. ^63^	N.A.
*Tfcp2l1:* 5’-AGGTGCTGACCTCCTGAAGA; 5’- CAGGCT GTT ATCCCCACT GT	Wang et al. ^64^	N.A.
*Gapdh*: 5’-CAAGGCTGTGGGCAAGGTCATCC; 5’- CTCCAGGCGGCAGGTCAGATCC	This study	N.A.
Software and algorithms		
ImageJ	Schindelin et al. ^65^	N.A.
Matlab R2020a	Natick, Massachusetts: The MathWorks Inc	N.A.
Leica LASX	Leica	N.A.

## Data Availability

This paper analyzes existing, publicly available scRNA-seq data. These accession numbers for the datasets are listed in the key resources table. The script for scRNA-seq analysis, network reconstruction and clone mapping, has been deposited at GitHub and is publicly available. DOIs are listed in the key resources table. Any additional information required to reanalyze the data reported in this paper is available from the lead contact upon request.
