# Supplementary material for: A cellular hierarchy of Notch and Kras signaling controls cell fate specification in the developing mouse salivary gland
Source: Dev Cell. Author manuscript; Available in PMC 2023 Aug 4. (PMC7614884; doi:10.1016/j.devcel.2022.12.009)
Supplement: Supplemental Tables and Figures [file EMS182036-supplement-Supplemental_Tables_and_Figures.pdf]

effect between different stages was then removed by using RunHarmony function of harmony (v1.0) with default parameters [72]. The batch-corrected data was used for non-linear dimension reduction based on t-distributed stochastic neighbor embedding (TSNE) implemented in runTSNE function (default parameter used) of R package scater (v1.10.1) [73].

Cell type annotation and calculation of marker genes: We then performed Louvain clustering (k-nearest neighbor = 7) for the data without cell cycle effect, which resulted in 12 clusters. The 12 clusters were then classified into the following 6 cell states based on known marker genes shown in Figure S5C: endbud, myoepithelial, basal duct, proacinar, and luminal duct state, as well as cells undergoing epithelial-to-mesenchymal transition (EMT). The EMT cells were filtered out for further analysis. Then, we identified highly expressed (a.k.a. marker) genes satisfying a given criterion (i.e., FDR < 0.01, log2(fold-change) > 0.445) for each cell state compared to others at E16 using findMarkers function of scran with default parameters, as cells at E14 are not differentiated enough to identify the marker genes (Table S4).

Pseudotime analysis: We then aimed to infer a potential lineage relationship between the putative multipotent progenitor population and bipotent and unipotent progenitor lineages. To this end, we performed pseudotime analysis using the R package monocle (v2.18.0) for the epithelial cells at E14 and E16 [74]. First, based on the marker genes above, dimension reduction was carried out by the DDRTree algorithm implemented in monocle. Next, the cells were ordered using orderCells function of monocle with default parameters to calculate pseudotime along lineage specification. As a result, 3 bifurcation points and 7 cell states were identified (Figure 3A). Based on the cell states, marker genes were calculated using the findMarkers function (FDR < 0.01 and log2(fold-change) > 0.5) with the batch effect from the different stages blocked. We then displayed the expression profiles for the marker genes along the pseudotime for each of four trajectories such as myoepithelial, basal duct, proacinar and luminal duct lineage (Figure 3E-H, Table S5). For each gene, auto-scaled gene expression was plotted using a rolling mean along its trajectory with a window size of 10% of cells. On the other hand, based on experimental clues, the averaged expression of downstream genes of Kras and Notch pathways were mapped along the pseudotime trajectories to infer the pathway activity along the fate specification. The downstream genes used for Notch were: *Hes1*, *Hey1*, *Notch1*, *Lfng*, *Nrap*, *Heyl* and *Maml1*. The downstream genes for Kras were *Fgfr1*, *Fgfr2*, *Spry1*, *Spry2*, *Etv1*, *Etv4* and *Etv5*.

The results generated here by pseudotime do not agree fully with the hierarchical organization proposed by Hauser et al., where differentiation into basal, myoepithelial and acinar lineages were thought to progress through luminal ductal progenitors. In this context, we would note that, in contrast to Hauser et al., we placed emphasis on data from the early embryonic stages and did not include data from postnatal and adult stages, which were beyond the scope of our tracing studies. It may be that this focus provides a finer resolution of the pattern of lineage restriction that becomes compromised when data is integrated across the broad range of developmental times.

Supplemental video

Movie S1. 3D view of the Figure 1E, Related to Figure 1.

Movie S2. Z-stack of a *RosaCreERT;Confetti* clone induced at E13.5 and traced for up to E18.5, Related to Figure 1.

Supplemental Tables

Table S1. Raw data of Figure 1G-H, Figure 4C-D, G-H, Figure 5O-P, Figure 6F-G, Figure S3C, Figure S6E-F and Figure S7F, Related to Figure 1.

Table S4. List of marker gene expression per cell type of the integrated E14.5, E16.5 map in Figure S5D, Related to STAR Methods.

Table S5. Average expression of genes along the pseudotime maps in Figure 3E-H, Related to Figure 3.

References

1. Auperin, A. (2020). Epidemiology of head and neck cancers: an update. Curr Opin Oncol *32*, 178-186. 10.1097/CCO.0000000000000629.
2. Mendez, L.C., Moraes, F.Y., Poon, I., and Marta, G.N. (2016). The management of head and neck tumors with high technology radiation therapy. Expert Rev Anticancer Ther *16*, 99-110. 10.1586/14737140.2016.1121111.
3. Rocchi, C., and Emmerson, E. (2020). Mouth-Watering Results: Clinical Need, Current Approaches, and Future Directions for Salivary Gland Regeneration. Trends Mol Med *26*, 649-669. 10.1016/j.molmed.2020.03.009.
4. Lombaert, I.M., Knox, S.M., and Hoffman, M.P. (2011). Salivary gland progenitor cell biology provides a rationale for therapeutic salivary gland regeneration. Oral Dis *17*, 445-449. 10.1111/j.1601-0825.2010.01783.x.
5. Patel, V.N., and Hoffman, M.P. (2014). Salivary gland development: a template for regeneration. Semin Cell Dev Biol *25-26*, 52-60. 10.1016/j.semcdb.2013.12.001.
6. Borghese, E. (1950). The development in vitro of the submandibular and sublingual glands of Mus musculus. J Anat *84*, 287-302.
7. Tucker, A.S. (2007). Salivary gland development. Semin Cell Dev Biol *18*, 237-244. 10.1016/j.semcdb.2007.01.006.
8. Knosp, W.M., Knox, S.M., and Hoffman, M.P. (2012). Salivary gland organogenesis. Wiley Interdiscip Rev Dev Biol *1*, 69-82. 10.1002/wdev.4.
9. Aure, M.H., Symonds, J.M., Mays, J.W., and Hoffman, M.P. (2019). Epithelial Cell Lineage and Signaling in Murine Salivary Glands. J Dent Res *98*, 1186-1194. 10.1177/0022034519864592.
10. Chatzeli, L., Gaete, M., and Tucker, A.S. (2017). Fgf10 and Sox9 are essential for the establishment of distal progenitor cells during mouse salivary gland development. Development *144*, 2294-2305. 10.1242/dev.146019.
11. Song, E.C., Min, S., Oyelakin, A., Smalley, K., Bard, J.E., Liao, L., Xu, J., and Romano, R.A. (2018). Genetic and scRNA-seq Analysis Reveals Distinct Cell Populations that

Supplemental Tables

Table S2, Related to Figure 2. Identity of clones sharing a common ancestor. U: unipotent, B: bipotent, T: tripotent, Q: quadpotent, A: acinar, L: luminal, B: basal, M: myoepithelial.

Level of common ancestor	Clones sharing the same common ancestor	Potency per clone	Number of cells per clone	Cell types in clone 1	Cell types in clone 2	Cell types in clone 3
3	2	[B,B]	[108,21]	[A;L]	[A;L]	
4	2	[U,T]	[21,154]	L	[A;L;M]	
4	2	[Q,T]	[86,84]	[A;L;B;M]	[A;L;M]	
6	3	[B,B,B]	[32,24,55]	[A;L]	[A;M]	[A;L]
6	2	[B,T]	[84,35]	[A;L]	[A;L;B]	
7	2	[B,U]	[237,4]	[A;L]	B	
7	2	[B,U]	[133,13]	[A;B]	L	
8	3	[U,B,T]	[239,65,303]	A	[A;L]	[A;L;M]
12	2	[B,B]	[14,22]	[A;B]	[A;L]	
12	2	[B,U]	[8,8]	[A;L]	L	

Table S3, Related to Figure 2 and Methods. Estimation of the number of uni bi and multipotent progenitors contributing to each compartment based on the fraction of clones of each type and their relative contribution to the overall cell count in the corresponding subtree (see Methods for details), for each of the n=3 repeats. S: Sample, N: Total number of clones contributing to the compartment, fu: Fraction of unipotent clones, fb: Fraction of bipotent clones, fm: Fraction of multipotent clones, cu: Relative contribution of unipotent clones, cb: Relative contribution of bipotent clones, cm: Relative contribution of multipotent clones, Nc: Number of progenitors contributing to the compartment, fu*Nc: Number of unipotent progenitors, fb*Nc: Number of bipotent progenitors, fm*Nc: Number of multipotent progenitors.

**Fraction of unipotent clones (fu)**	**Fraction of bipotent clones (fb)**	**Fraction of unipotent clones (fm)**	**Relative contribution of unipotent clones (cu)**	**Relative contribution of unipotent clones (cb)**	**Relative contribution of unipotent clones (cm)**	**Number of progenitors contributing to the compartment (Nc)**	**Number of unipotent progenitor s (fu*Nc)**	**Number of biipotent progenitor s (fb*Nc)**	**Number of multipotent progenitors (fm*Nc)**
0.3176	0.4118	0.2706	0.0183	0.0077	0.0050	97 (14)	30.7	39.9	26.2
0.2466	0.4521	0.3014	0.0123	0.0089	0.0054	116 (15)	28.5	52.2	34.8
0.1176	0.2941	0.5882	0.0076	0.0130	0.0060	122 (55)	14.3	35.8	71.5
0.0714	0.1786	0.7500	0.1655	0.0107	0.0081	51 (59)	3.6	9.0	37.9

Supplemental Figures

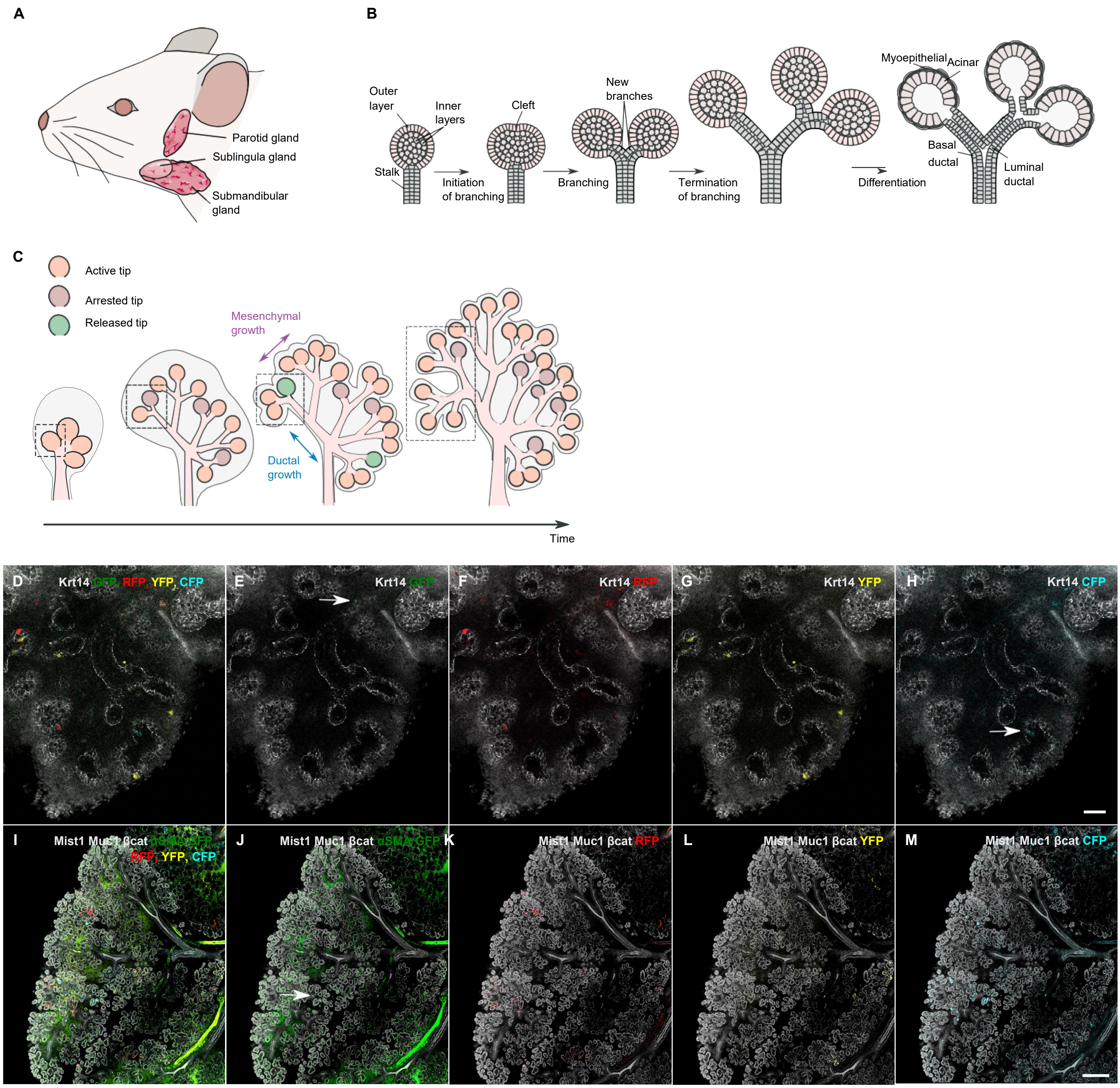

Figure S1. Schematic of the salivary gland development programme, Related to Figure 1. (A) Salivary gland types in mouse. (B) Schematic of salivary gland development and differentiation. (C) The model of the branching-arrested random walk on an expanding domain. (D-M) Clonal labelling at E14.5 after 1 day of tracing (D-H) and after 5 days of tracing at E18.5 (I-M) with *RosaCre-Confetti*. Scale bars are 50μm for D-G and 200 μm for I-M.

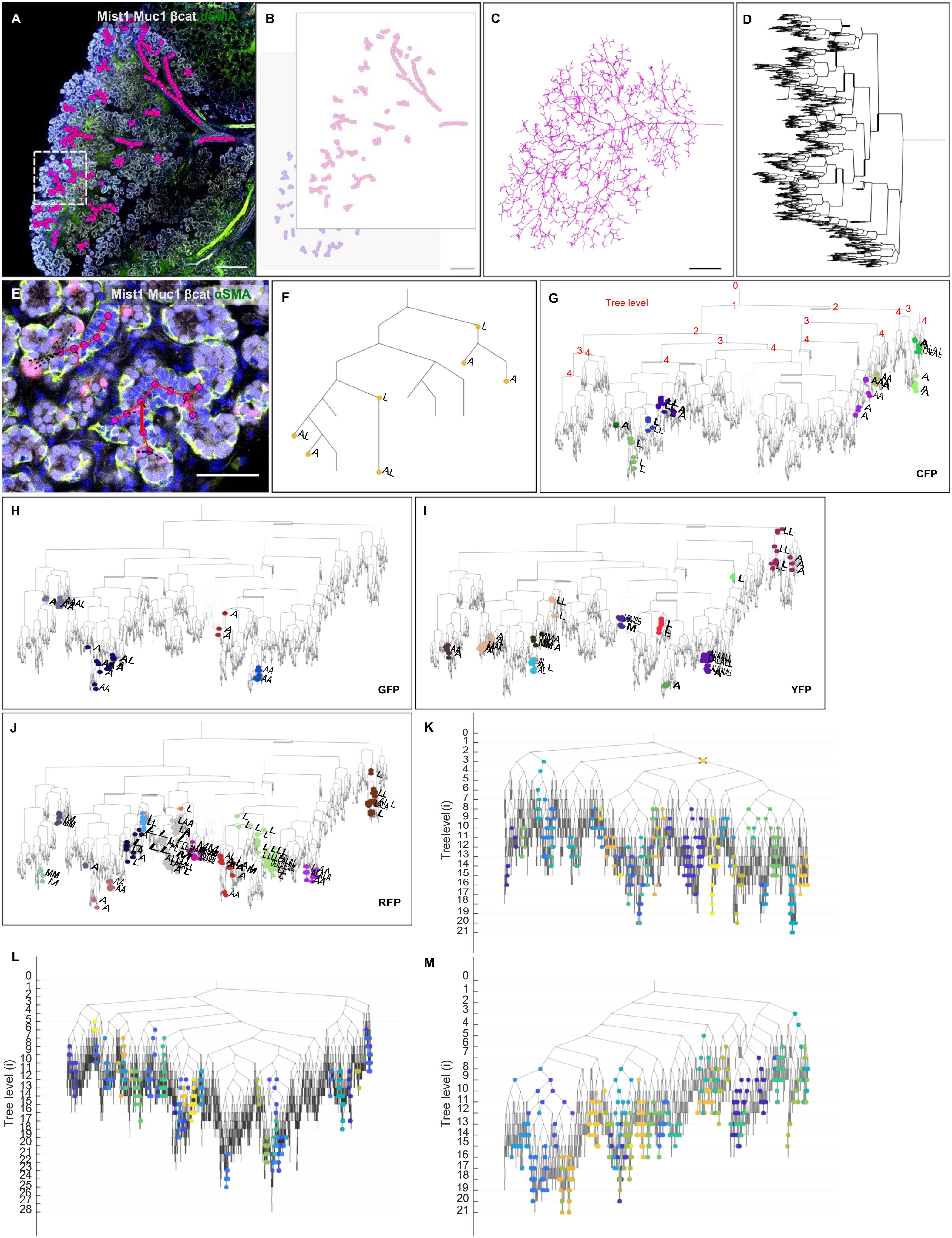
A
Mist1 Muc1 βcat αSMA
B
C
D
E
Mist1 Muc1 βcat αSMA
F
G
Tree level
CFP
H
GFP
I
YFP
J
RFP
K
Tree level (i)
L
Tree level (i)
M
Tree level (i)

Figure S2. Clonal segmentation and additional clone maps, Related to Figure 1 and Methods. (A-D) The ductal network of the first lobe of an E18.5 *RosaCre-Confetti* submandibular gland was segmented manually in 3D (C) (purple lines in A) by tracing the ducts through the entire thickness of the lobe (B) The network topology when then reduced in 2D (D). (E-F) Example of clonal mapping and ductal reconstruction in higher magnification. Red line with nodes indicates the ducts reconstructed from manual tracing. Black dotted lines show how labelled cells were assigned to a specific node in the ductal network. Green: αSMA, grey: Mist1, Muc1 and β-cat, red: RFP and blue: DAPI. Scale bar: 50µm. (F) A magnified view of a subtree at the terminal level. Yellow circular markers indicate the position of the subclones, indicating acinar (A) and luminal (L) cell type of each subclone. (G-J) Clones mapped on the ductal network shown in Figure 1F, split by the color of the fluorescent labelling. G shows CFP clones, H shows GFP clones, I shows YFP and J shows RFP clones. Here, each bifurcation represents a branching point in the network and vertical lines the ducts, the vertical lengths of which reflect the real duct length (G). (K) Reduced version of G-J maps where all the clones are mapped in the same tree. Here the ductal length is disregarded so that each bifurcation point was aligned. Non-renewing clones found on individual branches and marked with a red x. (L-M) The clone maps of the additional two biological replicates at E18.5.

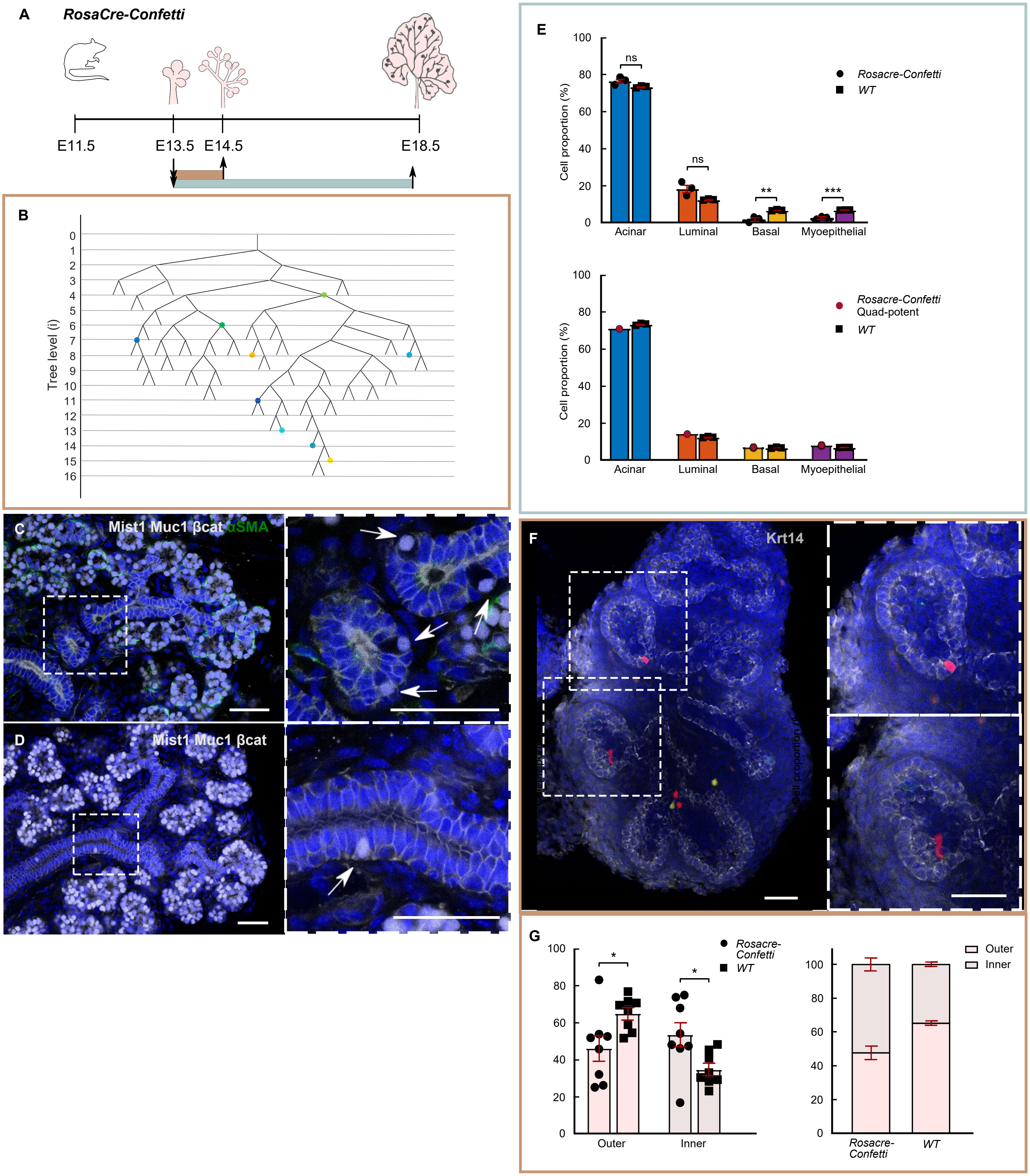

A
RosaCre-Confetti
E11.5
E13.5
E14.5
E18.5
B
Tree level (i)
0
1
2
3
4
5
6
7
8
9
10
11
12
13
14
15
16
C
Mist1 Muc1 βcat αSMA
D
Mist1 Muc1 βcat
E
Cell proportion (%)
ns
ns
**
***
Rosacre-Confetti
WT
Acinar
Luminal
Basal
Myoepithelial
Rosacre-Confetti
Quad-potent
WT
F
Krt14
G
Rosacre-Confetti
WT
Outer
Inner
*
*

Figure S3. Labelling with *RosaCre-Confetti* at E13.5 shows a small bias for inducing inner layer cells, Related to Methods. (A) Experimental time line of unbiased lineage tracing. *RosaCreERT;Confetti* (*RosaCre-Confetti*) mice were clonally induced at E13.5 and the submandibular glands were collected at E14.5 (brown line) or E18.5 (green line). (B) Clones mapped on the branching tree of the first lobe of an E14.5 *RosaCre-Confetti* mouse induced at E13.5 and traced up to E14.5 (brown bounding box). Colored markers indicate the location of subclones. (C-D) Acinar cells found on the luminal side of lower level ducts at E18.5 (J-J') and E16.5 (K-K'). Boxes outline the magnified images of J and K. Arrows pointing at the ductal acinar cells. Green: αSMA, grey: Mist1, Muc1 and β-cat and blue: DAPI. Scale bars 50µm. (E) Up: Proportion (mean ±SEM) of labelled acinar, luminal ductal, basal ductal and myoepithelial cells with *RosaCre-Confetti* at E18.5 after receiving tamoxifen at E13.5 (green bounding box). The proportion of acinar, luminal, basal and myoepithelial cells was calculated for the whole first lobe for each *RosaCre-Confetti* and was used as wild type (*WT*) control. Statistical analysis was performed with unpaired t-test. Ns: non-significant, *: $P \leq 0.05$, ***: $P \leq 0.001$. n = 3 experimental mice. See also Table S1, "unfiltered". Down: Proportion (mean) of labelled acinar, luminal ductal, basal ductal and myoepithelial cells with *RosaCre-Confetti* produced from quadpotent clones at E18.5 after receiving tamoxifen at E13.5 (green bounding box). (F) An E14.5 *RosaCre-Confetti* submandibular gland induced at E13.5 and traced up to E14.5 (brown bounding box). Top box: magnified image of C showing labelled cells at the outer Krt14+ layer. Bottom box: magnified image of C showing labelled cells at the inner Krt14- layer. Grey: Keratin 14 (Krt14), red: RFP, yellow: YFP, blue: DAPI. Boxes outline the magnified area in C' and C''. Scale bars: 50µm. (G) Left: proportion (mean ±SEM) of cells labelled at the outer and at the inner layer with *RosaCre-Confetti* at E14.5 after receiving tamoxifen at E13.5 (green bounding box). The proportion of cells located at the outer (Krt14+) and the inner layer (Krt14-) within wild type unlabelled buds was used as a wild type (WT) control. Statistical analysis was performed with unpaired t-test. *: $P \leq 0.05$. n = 8 experimental mice. Right: proportion of outer and inner labelled cells from all the experimental replicates ±SEP.

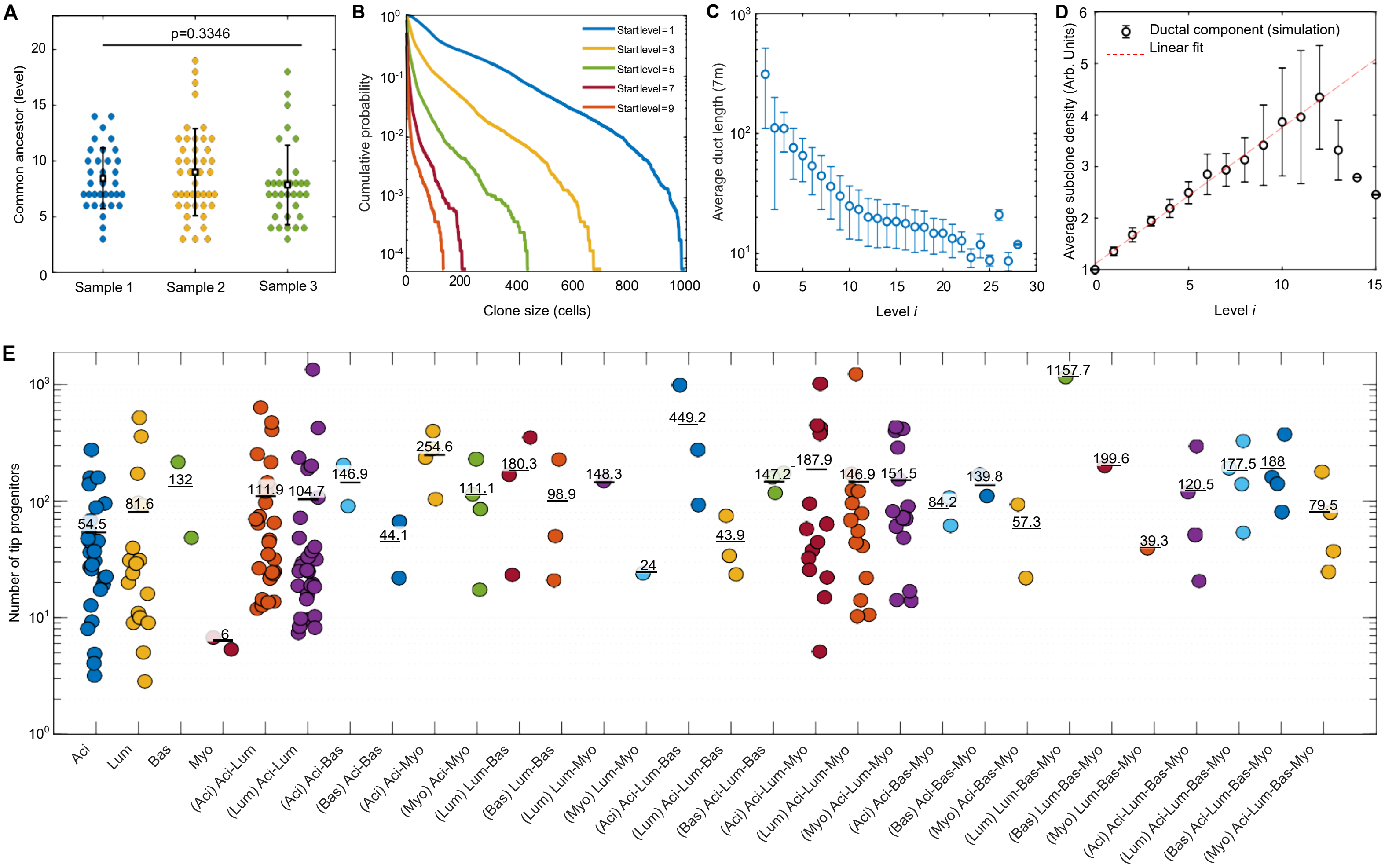

Figure S4. Additional data for the common ancestor plots, Related to Figure 2. (A) Level of the common ancestors of clones taken from the three experimental replicates at E18.5 (mean ±SD). Statistical analysis was performed using a two sample Kolmogorov-Smirnov test. (B) Numerical simulations showing correlation between size distribution and the level of common ancestor. (C) Average duct length ±SD as a function of branch level at E18.5. (taken from Bordeu et al., unpublished). (D) Numerical simulation showing a linear increase of the average subclone density ±SD as a function of level. (E) Estimates of the number of tip progenitors before taking into account the respective contribution of progenitor subtypes.

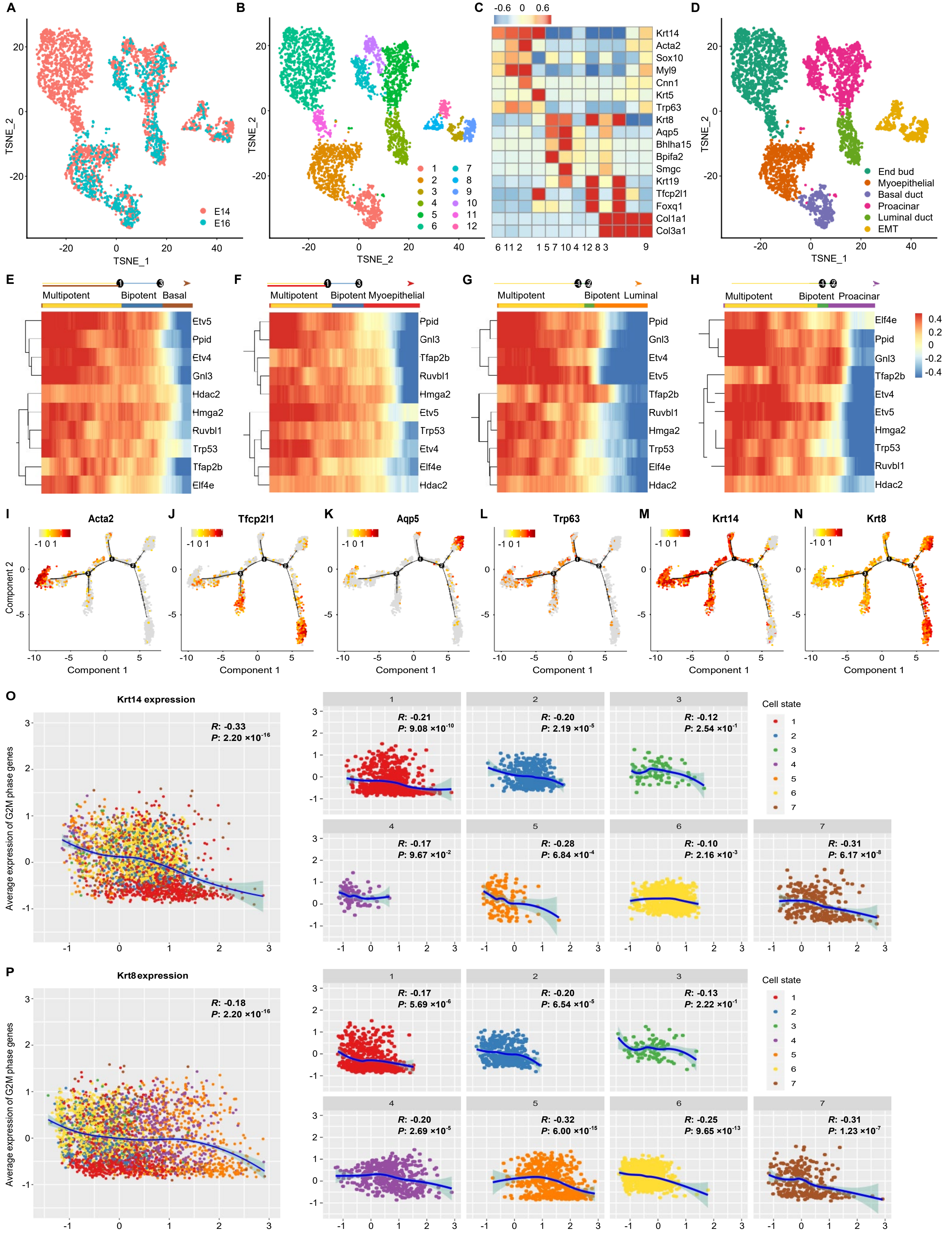

A
B
C
D
TSNE_2
TSNE_1
E14
E16
1
2
3
4
5
6
7
8
9
10
11
12
-0.6 0 0.6
Krt14
Acta2
Sox10
Myl9
Cnn1
Krt5
Trp63
Krt8
Aqp5
Bhlha15
Bpifa2
Smgc
Krt19
Tfcp2l1
Foxq1
Col1a1
Col3a1
6 11 2 1 5 7 10 4 12 8 3 9
End bud
Myoepithelial
Basal duct
Proacinar
Luminal duct
EMT
E
F
G
H
Multipotent
Bipotent
Basal
Myoepithelial
Luminal
Proacinar
Etv5
Ppid
Etv4
Gnl3
Hdac2
Hmga2
Ruvbl1
Trp53
Tfap2b
Elf4e
0.4
0.2
0
-0.2
-0.4
I
J
K
L
M
N
Acta2
Tfcp2l1
Aqp5
Trp63
Krt14
Krt8
-1 0 1
Component 2
Component 1
O
Krt14 expression
R: -0.33
P: 2.20 ×10-16
Average expression of G2M phase genes
R: -0.21
P: 9.08 ×10-10
R: -0.20
P: 2.19 ×10-5
R: -0.12
P: 2.54 ×10-1
R: -0.17
P: 9.67 ×10-2
R: -0.28
P: 6.84 ×10-4
R: -0.10
P: 2.16 ×10-3
R: -0.31
P: 6.17 ×10-8
Cell state
P
Krt8 expression
R: -0.18
P: 2.20 ×10-16
R: -0.17
P: 5.69 ×10-6
R: -0.20
P: 6.54 ×10-5
R: -0.13
P: 2.22 ×10-1
R: -0.20
P: 2.69 ×10-5
R: -0.32
P: 6.00 ×10-15
R: -0.25
P: 9.65 ×10-13
R: -0.31
P: 1.23 ×10-7

Figure S5. Additional data for the scRNA-seq analysis, Related to Figure 3 and Methods. (A) TSNE maps showing the distribution of epithelial cells at E14 and E16. (B) TSNE maps showing the Louvain clustering result (k-nearest neighbour = 7). (C) Heatmap representing expression of key marker genes per cell cluster in (B). Color gradient: scaled normalised log_2(UMI+1). (D) TSNE maps showing annotated cell types after batch correction. (E-H) Heatmaps showing the changes in the expression of genes highly expressed at state 6 (multipotent state) and downregulated along each of the trajectories from the multipotent progenitor to the following 4 sub-lineages: basal duct (K), myoepithelial cell (L), luminal duct (M), proacinal cell (N). Color bar on top: cell state. The names of key genes denoted on the right. Our selection criteria were transcription factors and genes involved in DNA remodelling and translation. Color gradient on bottom: scaled normalised log2(UMI+1). (I-N) Monocle maps representing expression of key marker genes such as *Acta2* (myoepithelial), *Tfcp2l1* (ductal), *Aqp5* (acinar), *Trp63* and *Krt14* (basal duct) and *Krt8*. Color gradient: log_{10}(UMI+0.1). (O-P) Scatter plots showing expression of cell cycle (G2M phase) genes vs. *Krt14* (K) or *Krt8* (L). The distributions of the data are displayed for all clusters (left) or each cluster (right) of Figure 3A. Expression values of x-y axis, shown as normalised log2(UMI+1) values, were scaled. The expression of G2M phase genes were averaged to displayed on y-axis. Blue curve and shade show fitting using loess method (curve) and 95% confidence level interval (shade), respectively. R, Pearson correlation coefficient. P, p-value of correlation test. The genes used for the G2M phase are as follows: *Hmgb2*, *Cdk1*, *Nusap1*, *Ube2c*, *Birc5*, *Tpx2*, *Top2a*, *Ndc80*, *Cks2*, *Nuf2*, *Cks1b*, *Mki67*, *Tmpo*, *Cenpf*, *Tacc3*, *Smc4*, *Ccnb2*, *Ckap2l*, *Ckap2*, *Aurkb*, *Bub1*, *Kif11*, *Anp32e*, *Tubb4b*, *Gtse1*, *Kif20b*, *Hjurp*, *Cdca3*, *Cdc20*, *Ttk*, *Cdc25c*, *Kif2c*, *Rangap1*, *Ncapd2*, *Dlgap5*, *Cdca2*, *Cdca8*, *Ect2*, *Kif23*, *Hmmr*, *Aurka*, *Psrc1*, *Anln*, *Lbr*, *Ckap5*, *Cenpe*, *Ctcf*, *Nek2*, *G2e3*, *Gas2l3*, *Cbx5*, *Cenpa*.

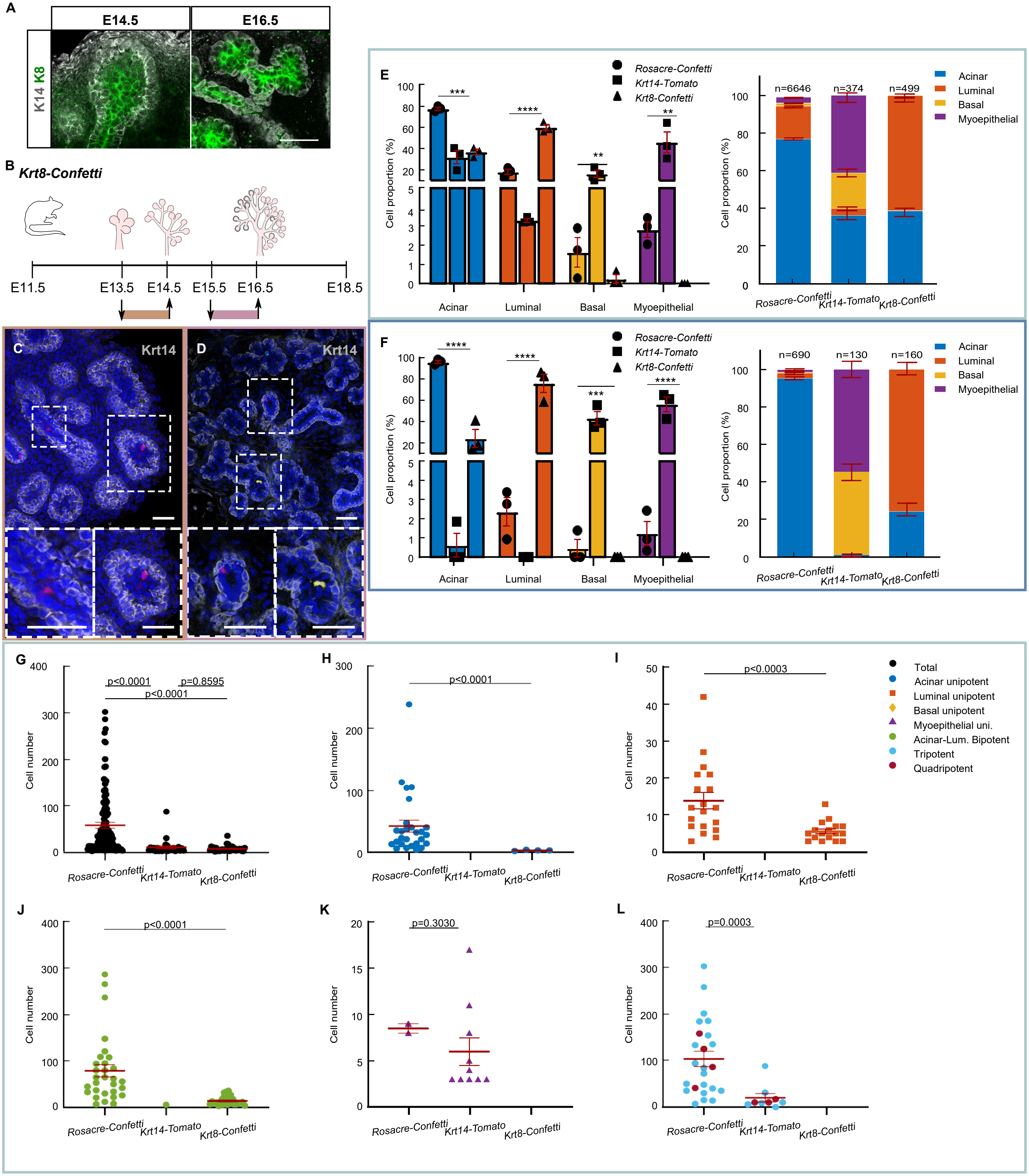
A
E14.5
E16.5
K14 K8
B
Krt8-Confetti
E11.5
E13.5
E14.5
E15.5
E16.5
E18.5
C
Krt14
D
Krt14
E
Rosacre-Confetti
Krt14-Tomato
Krt8-Confetti
Cell proportion (%)
***
****
**
**
Acinar
Luminal
Basal
Myoepithelial
n=6646
n=374
n=499
Acinar
Luminal
Basal
Myoepithelial
F
Rosacre-Confetti
Krt14-Tomato
Krt8-Confetti
Cell proportion (%)
****
****
***
****
Acinar
Luminal
Basal
Myoepithelial
n=690
n=130
n=160
G
Cell number
p<0.0001
p=0.8595
p<0.0001
Rosacre-Confetti
Krt14-Tomato
Krt8-Confetti
H
p<0.0001
I
p<0.0003
Total
Acinar unipotent
Luminal unipotent
Basal unipotent
Myoepithelial uni.
Acinar-Lum. Bipotent
Tripotent
Quadripotent
J
p<0.0001
K
p=0.3030
L
p=0.0003

Figure S6. Additional data for the lineage tracing with the *Krt8creERT;Confetti* and *Krt14creERT;Tomato*, Related to Figure 4. (A) Immunofluorescence for Krt14 (grey) and Krt8 (green) at an E14.5 and E16.5 wild type submandibular gland. Scale bar: 50μm. (B) Experimental timeline of targeted lineage tracing with *Krt8creERT;Confetti* (*Krt8-Confetti*). *Krt8-Confetti* mice were clonally induced either at E13.5 (brown line) or E15.5 (red line) and the submandibular glands were collected at either E14.5 or E16.5 respectively. (C) An E14.5 *Krt8-Confetti* submandibular gland induced at E13.5 and traced up to E14.5 (brown bounding box). Grey: Left box: magnified image of C showing labelled cells at the luminal K14- layer of the ducts. Right box: magnified image of C showing labelled cells at the inner K14- core of the endbud. Keratin 14 (Krt14), red: RFP, yellow: YFP, blue: DAPI. Boxes outline the magnified area in C' and C''. Scale bars: 50μm. (D) An E16.5 *Krt8-Confetti* submandibular gland induced at E15.5 and traced up to E16.5 (red bounding box). Left box: magnified image of D showing labelled cells at the luminal K14- layer of the ducts. Right box: magnified image of D showing labelled cells at the inner K14- core of the endbud. Grey: Keratin 14 (Krt14), red: RFP, yellow: YFP blue: DAPI. Scale bars: 50μm. (E) Left: proportion (mean ±SEM) of labelled acinar, luminal ductal, basal ductal and myoepithelial cells taken from *RosaCreERT;Confetti* (*Rosacre-Confetti*), *Krt14creERT;Tomato* (*Krt14-Tomato*) and *Krt8creERT;Confetti* (*Krt8-Confetti*) at E18.5 after receiving tamoxifen at E13.5 for each biological replicate (green bounding box). Due to lower induction with the *Krt14-Tomato* and *Krt8-Confetti*, 3 salivary glands were pooled to make 1 biological replicate. Statistical analysis was performed using one way Anova. ***: P ≤ 0.001, ****: P ≤ 0.0001. n = 3 biological replicates. Right: proportion of labelled acinar, luminal ductal, basal ductal and myoepithelial cells taken from E from all the biological replicates. n = number of cells. (F) Left: proportion (mean ±SEM) of labelled acinar, luminal ductal, basal ductal and myoepithelial cells taken from *RosaCreERT;Confetti* (*Rosacre-Confetti*), *Krt14creERT;Tomato* (*Krt14-Tomato*) and *Krt8creERT;Confetti* (*Krt8-Confetti*) at E18.5 after receiving tamoxifen at E15.5 for each biological replicate (blue bounding box). Due to lower induction with the *Krt14-Tomato* and *Krt8-Confetti*, 3 salivary glands were pooled to make 1 biological replicate. Statistical analysis was performed using one way Anova. ***: P ≤ 0.001, ****: P ≤ 0.0001. n = 3 biological replicates. Right: proportion of labelled acinar, luminal ductal, basal ductal and myoepithelial cells taken from E from all the biological replicates. n = number of cells. (G-L) Cell number (mean ±SEM) of all the clones (G), of acinar unipotent (H), luminal unipotent (I), acinar-luminal bipotent (J), myoepithelial unipotent (K) and multipotent clones (L) in *Rosacre-Confetti*, *Krt14-Tomato* and *Krt8-Confetti* induced from E13.5-E18.5 (green bounding box). Statistical analysis was performed using Mann Whitney test. Ns: non-significant, ***: P ≤ 0.001, ****: P ≤ 0.0001. n = clones. n = 112 total number of clones in *Rosacre-Confetti*, 33 clones in *Krt14-Tomato* and 49 clones in *Krt8-Confetti*.

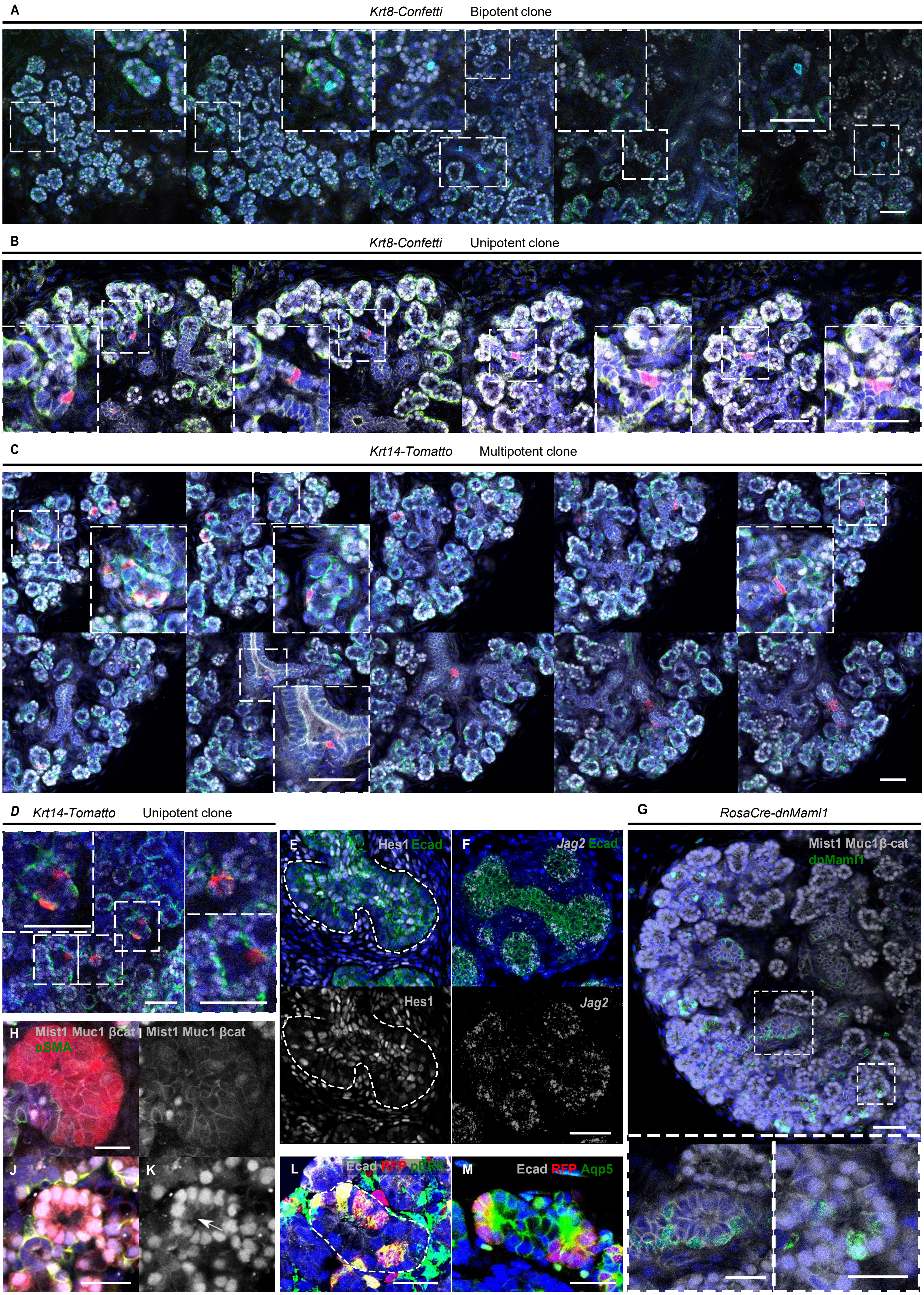

A
Krt8-Confetti Bipotent clone
B
Krt8-Confetti Unipotent clone
C
Krt14-Tomatto Multipotent clone
D
Krt14-Tomatto Unipotent clone
E
Hes1 Ecad
Hes1
F
Jag2 Ecad
Jag2
G
RosaCre-dnMaml1
Mist1 Muc1β-cat
dnMaml1
H
Mist1 Muc1 βcat
αSMA
I
Mist1 Muc1 βcat
J
K
L
Ecad RFP pERK
M
Ecad RFP Aqp5

Figure S7. Additional data for the lineage tracing with the *Krt8-Confetti* and *Krt14-Tomato*, and additional data for Notch and Kras signalling, Related to Figure 4, 5 and 6. (A) A bipotent *Krt8-Confetti* clone induced from E13.5 and traced up to E18.5. Boxes outline the magnified area in A showing labelled acinar and luminal cells. (B) A unipotent *Krt8-Confetti* clone induced as described in A. Boxes outline the magnified areas in B showing labelled luminal cells. (C) A multipotent *Krt14-Tomato* clone induced as described in A. Boxes outline the magnified area in C showing labelled acinar, myoepithelial, luminal and basal ductal cells. (D) A unipotent *Krt14-Tomato* clone induced a described in A. Boxes outline the magnified areas in D showing labelled myoepithelial cells. Scale bars: 50μm. (E) Immunofluorescence for Hes1 and Ecad at an E14.5 wild type submandibular gland. Gray: Hes1, green: E-cad and blue: DAPI. Scale bars: 50μm. (F) *In situ* hybridization for *Jag2* and immunofluorescence for Ecad at an E15.5 wild type submandibular gland. Gray: *Jag2*, green: E-cad and blue: DAPI. Scale bars: 50μm. (G) Representative image of low Notch cells (green) produced by labelling at E13.5 with *RosaCre-dnMaml1*. Left: magnified image of G showing GFP basal ductal cells with low Notch. Right: magnified image of G showing a GFP labelled acinar cell with low Notch. Green: GFP, grey: Mist1, Muc1 and β-cat and blue: DAPI. Scale bars: 50μm and 25μm. (H-I) Low intensity RFP+ Mist1+ cells located at the apex of an acini and produced by the *RosaCre-Red2Kras* induced at E13.5 and traced up to E18.5. (J-K) Normally differentiated *RosaCre-Red2Kras* acinar cells produced as described in H. Green: αSMA, grey: Mist1, Muc1 and β-cat, red RFP and blue: DAPI. Scale bars: 25μm. (L-M) Acinar cells expressing *Red2Kras* have high Erk activation and can undergo normal differentiation. (L) Nuclear pErk localisation in RFP+ *Red2Kras* acinar cells. Dotted line outlines the acini. Green: pERK, grey: Ecad, red: RFP. (M) Expression of Aqp5 in RFP+ *Red2Kras* acinar cells. Green: Aqp5, grey: Ecad, red: RFP. Scale bars: 25μm.
